# A Pragmatic Approach to Assess the Exposure of the Honey Bee (*Apis mellifera*) When Subjected to Pesticide Spray

**DOI:** 10.1371/journal.pone.0113728

**Published:** 2014-11-20

**Authors:** Yannick Poquet, Laurent Bodin, Marc Tchamitchian, Marion Fusellier, Barbara Giroud, Florent Lafay, Audrey Buleté, Sylvie Tchamitchian, Marianne Cousin, Michel Pélissier, Jean-Luc Brunet, Luc P. Belzunces

**Affiliations:** 1 INRA, Laboratoire de Toxicologie Environnementale, UR 406 A&E, CS 40509, 84914 Avignon Cedex 9, France; 2 ANSES, French Agency for Food, Environmental and Occupational Health Safety, 27–31 Avenue du Général Leclerc, 94701 Maisons-Alfort, France; 3 INRA, UR Ecodéveloppement, CS 40509, 84914 Avignon Cedex 9, France; 4 Department of Diagnostic Imaging, CRIP, National Veterinary School (Oniris), Nantes, France; 5 Université de Lyon, Institut des Sciences Analytiques, UMR5280 CNRS Université Lyon 1, ENS-Lyon, 5 rue de la Doua, 69100 Villeurbanne, France; University of British Columbia, Canada

## Abstract

Plant protection spray treatments may expose non-target organisms to pesticides. In the pesticide registration procedure, the honey bee represents one of the non-target model species for which the risk posed by pesticides must be assessed on the basis of the hazard quotient (HQ). The HQ is defined as the ratio between environmental exposure and toxicity. For the honey bee, the HQ calculation is not consistent because it corresponds to the ratio between the pesticide field rate (in mass of pesticide/ha) and LD_50_ (in mass of pesticide/bee). Thus, in contrast to all other species, the HQ can only be interpreted empirically because it corresponds to a number of bees/ha. This type of HQ calculation is due to the difficulty in transforming pesticide field rates into doses to which bees are exposed. In this study, we used a pragmatic approach to determine the apparent exposure surface area of honey bees submitted to pesticide treatments by spraying with a Potter-type tower. The doses received by the bees were quantified by very efficient chemical analyses, which enabled us to determine an apparent surface area of 1.05 cm^2^/bee. The apparent surface area was used to calculate the exposure levels of bees submitted to pesticide sprays and then to revisit the HQ ratios with a calculation mode similar to that used for all other living species. X-tomography was used to assess the physical surface area of a bee, which was 3.27 cm^2^/bee, and showed that the apparent exposure surface was not overestimated. The control experiments showed that the toxicity induced by doses calculated with the exposure surface area was similar to that induced by treatments according to the European testing procedure. This new approach to measure risk is more accurate and could become a tool to aid the decision-making process in the risk assessment of pesticides.

## Introduction

Human activity generates many environmental disruptions and myriads of anthropogenic chemical substances [1]. These substances include plant protection products (PPPs), or pesticides, of which the main families used are herbicides, insecticides and fungicides [2]. As a general feature, chemical substances are subjected to a risk assessment for their effects on human and non-target organisms. The assessment procedure is based on the comparison between the environmental or individual levels of exposure to the substances and the toxicity of these substances, which correspond to the exposure thresholds from which adverse effects can occur in biological organisms exposed through different routes (contact, oral and inhalation) [3], [4]. Generally, the exposure thresholds used in risk assessment for acute effects are toxicological values such as the contact median Lethal Dose or Concentration (LD_50_ or LC_50_). For long-term studies, and in particular reprotoxic effects, the threshold values used are the No Observed (Adverse) Effect Level or Concentration (NOAEL, NOEL, NOAEC and NOEC), defined as the highest exposure level for which no (adverse) effects were observed. In human risk assessment a more modern approach involves the determination of benchmark doses (BMD) or concentrations, which correspond to the levels of exposure at which a given effect is observed, determined by modeling the entire dose-effect relationship [5], [6]. These toxicological values can be lowered by uncertainty or safety factors such as those used to take into account intraspecific variability and extrapolation from model species, which correspond to interspecific variability. From the exposure and toxicological values, a Hazard Quotient (HQ = Exposure/Toxicity) or Toxicity to Exposure Ratio (TER = Toxicity/Exposure) can be derived to assess the risk presented by chemicals or pesticides to human and non-target organisms. Hence, when the exposure level is higher than the toxicological value, the situation is at risk for the considered biological organism, which results in HQ or TER values higher or lower than 1, respectively.

For all organisms including humans, the HQ and TER are calculated on the basis of toxicological data obtained on individuals exposed through the pertinent routes of exposure. In the environment, the honey bee appears as an atypical case because both toxicological methods and risk assessment procedures are not always adapted to the environmental situation. For example, (i) the determination of the LD_50_ is relevant for acute contact during a treatment with the active substance, such as pesticide sprays. Although still performed until now, these data cannot be used to extrapolate the chronic contact toxicity. (ii) For residual contact with contaminated plants, no method exists in the registration procedure of pesticides. In the past, the tarsal contact test was used to assess the toxicity induced by repeated contact with a contaminated surface [7]. However, this test is no longer used today. (iii) No method exists to assess the toxicity elicited by inhalation. (iv) Acute oral LD_50_ values are relevant to assess the toxicity induced by an acute exposure to a pesticide through contaminated food, but are inadequate to determine the chronic oral toxicity [8]. The first laboratory approach to assess the oral chronic toxicity of pesticides was proposed in 2001 [9]. Today, chronic toxicity is being considered for inclusion in the pesticide registration procedure by the French Commission for the Biological Assays (CEB), the European Food Safety Agency (EFSA) and the Society of Environmental Toxicology & Chemistry (SETAC) [10]–[12]. (v) Up to now, risk assessment in honey bee was only based on acute exposure and not on chronic exposure, as practiced for other organisms, which explains the lack of experimental data [3], [4]. (vi) A critical step in the assessment of the risk presented by pesticides to bees is based on the determination of the HQ [11], [13]. For the HQ and TER, data of the same nature are compared, namely individual or environmental exposure levels and exposure levels below which no (adverse) effect can be observed, which results in HQ and TER values without units. However, for the honey bee, the contact HQ corresponds to the ratio of the pesticide field rate, expressed in mass/ha (generally g/ha), to LD_50_, expressed in mass/bee (generally µg/bee). This calculation method for the HQ is used because it is difficult to convert field rates of sprayed pesticides into doses to which honey bees are exposed. This results in the HQ with a unit corresponding to a number of bee per ha, which is not useful to properly assess the risk to bees. Thus, after its introduction by Atkins in 1981 [14], the contact HQ ratio was used empirically to assess the risk of pesticides to the honey bee and the values fluctuated between 25 [13] and 85 as the toxicological threshold [11].

In this study, we used a pragmatic approach to determine the exposure levels of bees subjected to a pesticide treatment in the field. We used experimental sprayings to estimate the apparent exposure surface area of a honey bee that enabled converting pesticide field rates into acute doses received by the bees. In a second step, the accuracy of the exposure surface area was probed by comparing the toxicity induced by spraying at given field rates with the toxicity induced by treatments, achieved according to the procedures of the European and Mediterranean Plant Protection Organization (EPPO) and the Organization for Economic and Co-operation Development (OECD) [15], [16], with doses calculated from field rates and the apparent exposure surface area. Finally, the exposures to which bees can be submitted after pesticide spraying were calculated and the hazard quotients were revisited.

## Materials and Methods

### Chemicals

Abamectin (Agrimec gold), acetamiprid (Supreme), chlorpyrifos-ethyl (Nelpon 480), cyfluthrin (Baythroïd), cypermethrin (Cyperfor S), deltamethrin (Decis protech), dimethoate (Danadim progress), esfenvalerate (Sumi-alpha), fenoxycarb (Insegar), hexythiazox (Nissorun), imidacloprid (Confidor), iprodione (Rovral WG), lambda-cyhalothrin (Karate xpress), pyriproxyfen (Admiral pro), tau-fluvalinate (Klartan), tebuconazole (Horizon arbo), thiacloprid (Calypso), and thiamethoxam (Flagship pro) were purchased from Escudier Christian SARL (Avignon, France). Clothianidin (Dantop 50 WG) and prochloraz (Octave) were obtained from Coopérative Agricole Provence Languedoc (Châteaurenard, France). Abamectin (97% pure), acetamiprid (98.1% pure), chlorpyrifos-ethyl (98.5% pure), clothianidin (99% pure), cyfluthrin (98% pure), cypermethrin (94% pure), deltamethrin (98% pure), dimethoate (98.5% pure), esfenvalerate (99% pure), imidacloprid (99% pure), lambda-cyhalothrin (98.5% pure), prochloraz (98% pure), tau-fluvalinate (94% pure), thiacloprid (98% pure), and thiamethoxam (98.5% pure) were purchased from TechLab. DMSO (Dimethyl sulfoxide) was obtained from Sigma-Aldrich.

### Honey bee collection

Honey bee workers (*Apis mellifera*) were collected from the reserve frames of the hive body from healthy colonies (≥30 000 individuals) that were carefully monitored for their sanitary state in the experimental apiary of the INRA Research Unit *Abeilles* & *Environnement* (*Bees* & *Environment*) of Avignon. After being anesthetized with CO_2_, bees were distributed in cages (10.5×7.5×11.5 cm) by groups of 30 individuals and placed in the dark in a thermostated chamber at 28±2°C and 60±10% RH. The ambient temperature of the thermostated chamber was chosen as the temperature preferred by the honeybees during the night [17], [18]. Bees were fed *ad libitum* with candy (Apifonda + powdered sugar) and water.

### Exposure to pesticide sprays

Bees were exposed by spraying in a Potter-type tower to the 20 different commercial pesticide preparations (Table 1) at application rates chosen to be as close as possible to field conditions. The amounts of active substances sprayed were selected to be less than or equal to the lowest registered dose. Twelve replicates of 30 bees were performed for each pesticide treatment. Bees were first anesthetized and put on a 200 cm^2^ Plexiglas disc subjected to rotation at a speed of 23 rpm to ensure a homogeneous deposit of the sprayed solution [19]. The deposit was previously calibrated to obtain a rate of 2.32±0.13 µL/cm^2^. In addition to the calibrations, the deposit was controlled just before, during and after each series of 12 replicates (experimental calibration). Immediately after spraying, the bees from 6 replicates were frozen at −80°C to fix the dose of pesticide they received. In parallel, the mortality was followed over time on the remaining 6 replicates by keeping the bees in the thermostated chamber.

**Table 1:** Summary of commercial products and their application rates used for the spraying treatment.

Effect	Class	Active substance	Commercial product	Application rate (g a.s./ha)
Fungicide	Dicarboximide	Iprodione	Rovral WG	600
	Imidazole	Prochloraz	Octave	230.5
	Triazole	Tebuconazole	Horizon arbo	75
Growth regulator	Carbamate	Fenoxycarb	Insegar	75
	Carboxamide	Hexythiazox	Nissorun	26
	Pyridine based	Pyriproxyfen	Admiral pro	25
Insecticide	Avermectin	Abamectin	Agrimec gold	2
	Neonicotinoid	Acetamiprid	Supreme	30
		Clothianidin	Dantop 50 WG	3
		Imidacloprid	Confidor	0.2
		Thiacloprid	Calypso	50
		Thiamethoxam	Flagship pro	5
	Organophosphate	Chlorpyrifos-ethyl	Nelpon 480	3
		Dimethoate	Danadim progress	5
	Pyrethroid	Cyfluthrin	Baythroïd	2.5
		Cypermethrin	Cyperfor S	3
		Deltamethrin	Decis protech	1.875
		Esfenvalerate	Sumi-alpha	2
		Lambda-cyhalothrin	Karate xpress	5
		Tau-fluvalinate	Klartan	48

g a.s./ha: gram of active substance per hectare.

### Topical exposure to pesticides

Preliminary studies were conducted to assess the mortality rate induced by each pesticide. For each substance, 6 repetitions were systematically performed. However, for the doses inducing a low toxicity, the number of repetitions was increased for an accurate determination of the mortality rates (Table S1). Bees were exposed to 15 out of the 20 active substances because it was not possible to determine a dose-mortality relationship for 5 active substances (fenoxycarb, hexythiazox, iprodione, pyriproxyfen, tebuconazole) that exhibited a very low toxicity (LD_50_≥100 000 ng a.s./bee). The active substances were diluted in DMSO, and the control groups were treated with pure DMSO. The bees were treated on the thorax according to the EPPO procedure recommended by the European Commission [15], [20], [21]. Mortality was checked 24, 48, 72 and 96 hours after the exposure, with 96 hours corresponding to the duration at which no significant evolution of mortality was observed for all the tested active substances.

### Quantification of 20 pesticides

For each of the 20 pesticides of interest, the active substance was quantified in 3 repetitions. For each repetition, 5 g of frozen honey bees were weighed in a 50 mL centrifuge tube, and 10 mL of acetonitrile, 3 mL of water, 3 mL of heptane, citrate QuEChERS salts and 30 g of steel balls were added for grinding for 4 min at the cadency of 1000 strokes per min using a Geno/Grinder from SPEX Sample Prep (Metuchen, USA). Then, the tube was centrifuged for 2 min at 5000 g, and 6 mL of the lower acetonitrile phase were taken and placed in a pre-prepared 15 mL PSA/C18 tube. This tube was immediately shaken by hand, vortexed for 10 sec and centrifuged for 2 min at 5000 g. Finally, 4 mL of the extract were taken and put in a 10 mL cone-ended glass centrifuge tube for evaporation until 50 µL remained. This remaining extract was kept at −18°C until analysis.

For abamectin, acetamiprid, clothianidin, fenoxycarb, hexythiazox, iprodione, imidacloprid, lambda cyhalothrin, prochloraz, pyriproxyfen, thiacloprid and thiamethoxam, the analysis was performed on a Waters 2695 series Alliance HPLC (Waters, Milford, MA) coupled to a triple quadrupole mass spectrometer Quattro from Micromass (Manchester, UK) equipped with a Z-spray electrospray interface (ESI). Data were processed with MassLynx 4.1. Electrospray ionization was performed in the positive mode. The chromatographic separations were performed on a Nucleodur Sphinx RP-C18 (50×2 mm, 1.8 µm) column from Macherey-Nagel with in-line filter “krudkatcher” 0.5 µm porosity (Phenomenex). The column oven temperature was set to 40°C; the flow rate was 300 µL/min. Samples were analyzed with the mobile phase (A) water with 0.3 mM of ammonium formate and 0.05% formic acid, and (B) methanol. Ten µL were injected in the 90/10 mobile phase (A)/acetonitrile.

For chlorpyrifos-ethyl, cyfluthrin, cypermethrin, deltamethrin, dimethoate, esfenvalerate, tau-fluvalinate and tebuconazole, the analysis was conducted with a GC-ToF, a 6890 Agilent gas chromatograph coupled to a Time of Flight (ToF) mass spectrometer GCT Premier from Waters. Data were processed with MassLynx 4.1. Chromatographic separation was performed on a 30 m×0.25 mm I.D., with a 0.25 µm film thickness DB-XLB capillary column (Agilent Technologies, Avondale, USA). Helium (purity 99.999%) was used as a carrier gas at a constant flow of 1 mL/min. Injections were performed in the splitless mode, and 1 µL in 90/10 acetonitrile/analyte protectant mix was injected. The mass spectrometer was operated in the electron impact mode (EI, 70 eV). Acquisition was performed in the full scan mode with a scan range of m/z 50–550. Calibration was performed using the calibration wizard, with heptacosa as the reference.

Matrix-matched calibration was used for quantification. In each batch, 6 calibration points were prepared and injected as described above, with concentrations ranging between 4 and 60 ng/g. Quantification was performed using QuanLynx 4.1.

### Physical surface of the honey bee

The surface area of the bees, excluding the wings, was determined by X-ray computed tomography. Imaging was performed on 6 dead bees with a µPET/CT Inveon device (Siemens Preclinical Solutions, Knoxville, TN). The computed tomography (CT) images were acquired with the following settings: X-ray source tube voltage at 40 keV with a constant 500 µA current during 320 ms and 2-degree rotation to generate images with 41 µm of voxel resolution. Three-D reconstructions were performed using COBRA software (Siemens Preclinical Solutions). Using ImageJ, the stacks of images were thresholded, and the regions of interest (ROI) were automatically drawn on binary images. Each ROI was checked, and the surface areas obtained were then summed for each of the resulting 768 slices. The surface area of the wings was measured on 50 bees. The 4 wings were cut at the base of the thorax and placed on a microscope slide and slip covered with soapy water. Pictures of wings were taken under a microscope; ImageJ software was used to delineate the wings and to calculate their surface area.

### Data analysis

Statistical analyses were performed with R software (2013). The variations of the deposit distributions were tested using a t-test comparing preliminary and experimental calibrations. Pearson’s correlation and a linear regression were used to compare the mortalities obtained by spray contamination and those obtained by the EPPO procedure. For each dose, the stabilization of the dose-mortality response over time was determined with Fisher’s exact tests. The mortality was considered stabilized when there were no differences between two consecutive days for all the tested doses.

### LD_50_ calculation

The BMD approach consists in the selection of the model that describes the data using appropriate model structures for the type of data (dichotomous or quantal). A mathematical model is applied to the experimental data to produce the dose-response curve with the best fit. Details of the full process on this approach are presented in the BMD Software technical guidance from the US EPA (http://www.epa.gov/ncea/bmds/). For the dichotomous (or quantal) data, the response or effect may be reported as either the presence or absence of an effect. The dose-response models describe how the probability or frequency of a specified response changes with the dose level (50%). The different models were classified by Akaike’s Information Criterion (AIC), and the model with the lowest AIC was chosen for the determination of LD_50_ for each active substance.

## Results

### Control of the active substance concentrations

The concentration of the working solutions of active substances and the 20 commercial pesticide formulations were controlled by chemical analysis (Table 2). The concentration variations of the formulations ranged between −25% (tebuconazole) and +38% (abamectin) of the concentration indicated by the manufacturer. This suggests that the technical process to adjust pesticide concentration is not accurately controlled by the manufacturer. The measured concentrations were used in all cases to determine the actual exposure levels of the bees after spraying.

**Table 2:** Comparison of the commercial and measured concentrations.

Active substance	Commercial concentration	Measured concentration		Difference (%)
		Mean	S.D.	
Abamectin	18 g/L	24.91 g/L	1.27 g/L	+38.4
Acetamiprid	200 g/kg	243.66 g/kg	6.14 g/kg	+21.8
Chlorpyrifos-ethyl	480 g/L	500.20 g/L	0.02 g/L	+4.2
Clothianidin	500 g/kg	453.71 g/kg	23.11 g/kg	−9.3
Cyfluthrin	50 g/L	46.45 g/L	0.09 g/L	−7.1
Cypermethrin	100 g/L	114.25 g/L	0.14 g/L	+14.2
Deltamethrin	15 g/L	14.77 g/L	0.72 g/L	−1.5
Dimethoate	400 g/L	433.35 g/L	0.92 g/L	+8.3
Esfenvalerate	25 g/L	25.10 g/L	0.50 g/L	+0.4
Fenoxycarb	250 g/kg	228.54 g/kg	14.49 g/kg	−8.6
Hexythiazox	104 g/kg	91.24 g/kg	2.94 g/kg	−12.3
Imidacloprid	200 g/L	230.29 g/L	12.24 g/L	+15.1
Iprodione	750 g/kg	794.34 g/kg	7.01 g/kg	+5.9
Lambda-cyhalothrin	50 g/kg	44.85 g/kg	2.47 g/kg	−10.3
Prochloraz	461 g/kg	391.69 g/kg	13.72 g/kg	−15
Pyriproxyfen	100 g/L	97.29 g/L	2.66 g/L	−2.7
Tau-fluvalinate	240 g/L	232.65 g/L	12.80 g/L	−3.1
Tebuconazole	250 g/kg	186.30 g/kg	10.32 g/kg	−25.5
Thiacloprid	480 g/L	448.97 g/L	0.07 g/L	−6.5
Thiamethoxam	10 g/L	10.25 g/L	0.16 g/L	+2.5

S.D.: Standard Deviation.

The concentrations of commercial products are expressed according to their nature, liquid or solid.

The quantification of the active substance was performed in triplicate from the phytopharmaceutical preparation used for the spray application.

The differences were expressed as percentages of the commercial concentrations.

### Calibration of the deposit in the spraying tower

**Figure 1:**
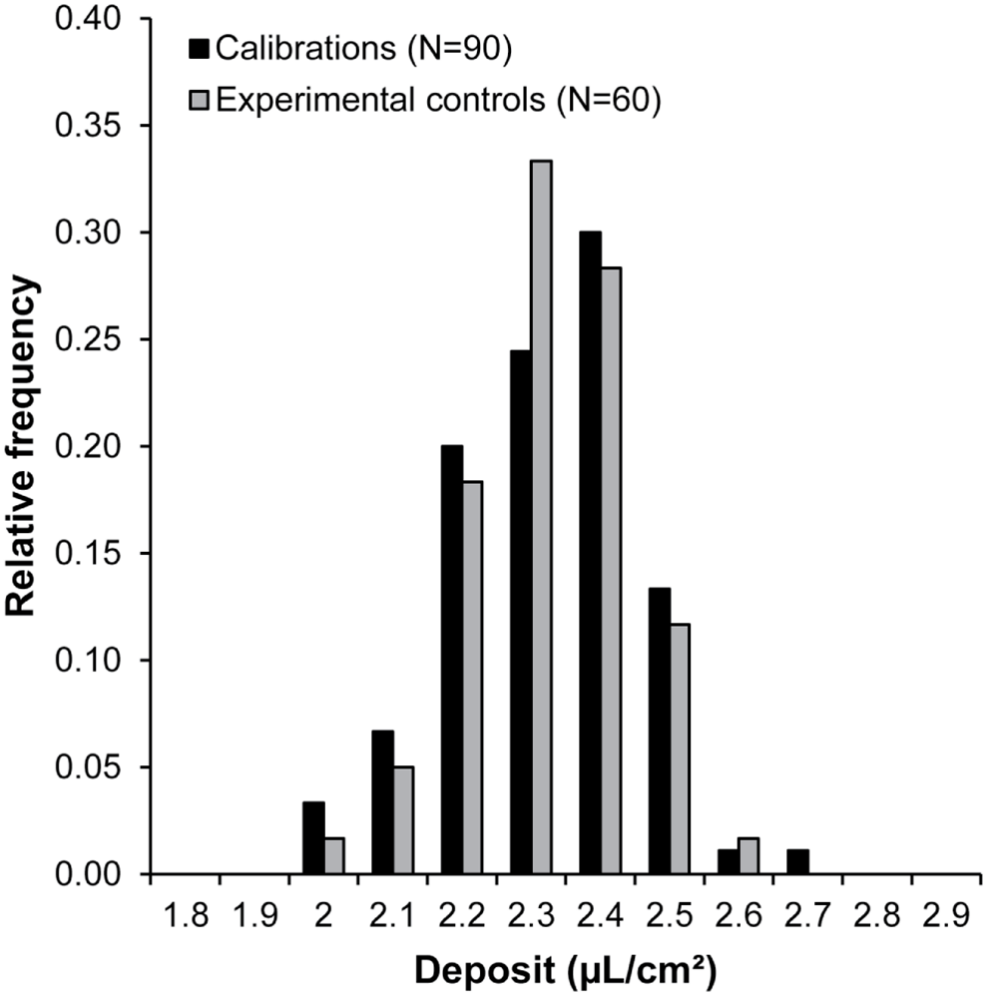
Distribution of the deposit during preliminary calibrations and experimental controls. The preliminary calibrations were performed before the experiment, and the experimental calibrations were performed during the experiment. The deposit was expressed in µL/cm^2^ of the disc. T-test preliminary vs. experimental calibrations; (t = 0.0739, df = 148, p-value = 0.9412).

To check the reliability of the deposit, preliminary calibrations and experimental controls were performed (Figure 1). No differences were found between the deposits performed during the calibration procedure and the experimental controls (t-test; t = 0.0739, df = 148, p-value = 0.9412). The mean values of the deposit for calibrations and experimental controls were 2.32±0.13 µL/cm^2^ and 2.32±0.11 µL/cm^2^, respectively (Table 3). These volumes corresponded to a field spraying volume of 232 L/ha, which was similar to volumes commonly used in agriculture [22].

**Table 3:** Comparison of preliminary calibrations and experimental controls of the deposit.

Calibration	Deposit (µL/cm^2^)		N_c_	C.I. 95%	Sprayed volume (L/ha)^a^	
	Mean	S.D.			Mean	S.D.
Preliminary	2.32	0.13	90	[2.30, 2.35]	232	13
Experimental	2.32	0.11	60	[2.30, 2.35]	232	11

S.D.: Standard Deviation.

Nc: Number of calibrations.

C.I. 95%: Confidence Interval at 95%.

a Sprayed volume corresponds to the deposit in field conditions.

### Determination of the exposure

Knowing the application rate of pesticides per surface area unit (*n* g/ha equal to 10×*n* ng/cm^2^) and the determination of the doses received per bee enabled estimating the apparent exposure surface area of a bee. For each pesticide, the application rate was corrected by the real deposit (see Table 4: Deposit of the experimental control). The dose received by the bees (in ng/g of bee) was determined by chemical analysis of residues. To avoid the degradation of pesticides, *in* *vivo* biotransformation was stopped by quick freezing of the bees still anesthetized at −80°C immediately after spraying. The mean mass of the bees was determined to calculate the dose received per individual (in ng/bee) (Table 4). The exposure surface area of a bee (in cm^2^/bee) is the ratio between the residues (in ng/bee) and the application rate (in ng/cm^2^). Considering all the pesticides, the mean apparent exposure surface area was 1.05±0.33 cm^2^/bee (n = 20).

**Table 4:** Determination of the exposure surface area per bee for each active substance.

Activesubstance	Deposit of theExperimentalcontrol(µL/cm^2^)		Correctedapplicationrate^a^(g a.s./ha)	Residues perbee^b^ (ng a.s./g)		Weight perbee (mg/bee)	Residues(ng a.s./bee)	Exposuresurfacearea^c^(cm^2^/bee)
	Mean	S.D.		Mean	S.D.			
Abamectin	2.35	0.05	2.8	261.1	44	112	29.2	1.04
Acetamiprid	2.43	0.10	38.2	1922.7	152	117	225.0	0.59
Chlorpyrifos-ethyl	2.48	0.10	3.3	263.1	7.1	121	31.8	0.95
Clothianidin	2.24	0.04	2.6	137.5	9.5	125	17.2	0.65
Cyfluthrin	2.20	0.11	2.2	313.5	43.4	113	35.4	1.61
Cypermethrin	2.45	0.04	3.6	335.6	22.5	126	42.3	1.17
Deltamethrin	2.32	0.04	1.8	112.7	4.9	122	13.7	0.75
Dimethoate	2.26	0.14	5.3	451.6	54.4	120	54.2	1.03
Esfenvalerate	2.31	0.08	2.0	186.5	37.8	122	22.8	1.14
Fenoxycarb	2.21	0.20	65.3	4393	40	122	536.0	0.82
Hexythiazox	2.36	0.10	23.2	3143	697	122	383.5	1.66
Imidacloprid	2.33	0.03	0.2	20.2	2	122	2.5	1.07
Iprodione	2.36	0.09	645.1	32980	1058	122	4023.6	0.62
Lambda-cyhalothrin	2.32	0.07	4.5	430	46	122	52.5	1.17
Prochloraz	2.28	0.05	192.7	13990	828	122	1706.8	0.89
Pyriproxyfen	2.24	0.14	23.4	2307	412	122	281.4	1.20
Tau-fluvalinate	2.44	0.09	48.9	5857	483	122	714.5	1.46
Tebuconazole	2.33	0.04	56.1	7370	157	122	899.1	1.60
Thiacloprid	2.32	0.08	46.7	3523.3	417.1	118	415.7	0.89
Thiamethoxam	2.27	0.05	5.0	279.7	14.5	119	33.3	0.66

S.D.: Standard Deviation (n = 3).

g a.s./ha: gram of active substance per hectare.

ng a.s./ha: nanogram of active substance per hectare.

ng a.s./bee: nanogram of active substance per bee.

a The application rate was corrected with the measured concentration (Table 3) and the experimental control deposit.

b This corresponds to the concentration of active substances found in the bees frozen just after contamination.

c The exposure surface area is the ratio between the residues (ng a.s./bee) and the corrected application rate (ng a.s./cm^2^).

For each treatment, the bees from the 6 replicates were counted, pooled, and weighed; the mean bee weight was determined by dividing the weight of bees by the actual number of bees.

### Spray toxicity vs. topical toxicity

**Figure 2:**
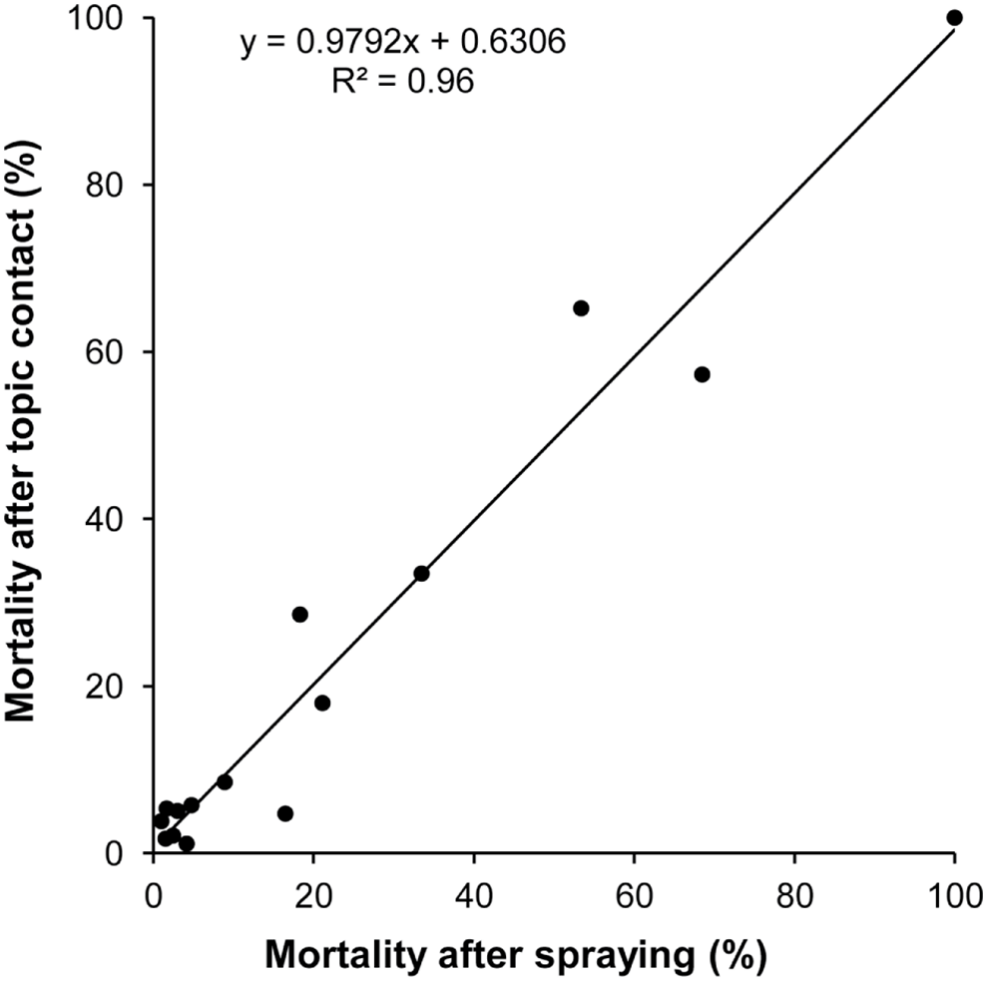
Correlation between mortality induced by spraying and mortality induced by topical treatment. Each dot represents one substance among the 15 tested on bees by thorax topical contact and spraying. The mortality elicited at 48 hours by spraying was given for the chosen field application rate (g a.s./ha) (Table 1). On the basis of the mean exposure surface area (1.05 cm^2^/bee), the application rate (g a.s./ha) was converted into an individual dose (ng a.s./bee) to treat the bees topically. The dose-mortality relationship at 48 hours was modeled for each of the 15 active substances, which enabled assessing the mortality that could be induced at a given field exposure.

To check the accuracy and reliability of the estimated mean exposure surface area of a bee for each formulation, the sprayed dose (in g/ha), which was associated with the corresponding mortality rate, was converted into an individual dose (in ng/bee) on the basis of an apparent surface area of 1.05 cm^2^/bee. In a second step, this individual dose was topically applied on the thorax according to the EPPO procedure. Then, the mortality rates obtained by spraying and topical application were compared by linear regression analysis (Figure 2). A good linear correlation between mortalities elicited by spraying and topical exposures was found (r^2^ = 0.960). It is noteworthy that the slope of the regression was very close to 1 (y = 0.9792x+0.6306), which showed that the mortality induced by topical exposure was not over- or underestimated compared with the response obtained by a spray exposure. The experimental data of the dose-mortality response obtained by topical application are presented in Figures S1, S2, S3, S4, S5, S6, S7, S8, S9, S10, S11, S12, S13, S14 and S15.

### Revision of the hazard quotient

The accuracy of the apparent exposure surface area previously estimated enabled calculating a new hazard quotient (HQ) based on the real exposure of the bees. Thus, the current HQ and revisited HQ were compared (Table 5). Currently, in bee toxicology, the HQ of a substance is defined as the ratio between the application rate (in g a.s./ha) and the LD_50_ expressed in µg a.s./bee [13]. The revisited HQ was based on the estimated exposure expressed in ng a.s./bee, calculated from the application rate and the exposure surface area per bee, and the LD_50_ was expressed in ng a.s./bee. This revisited HQ is a relative exposure level corresponding to the number of LD_50_ to which a bee is exposed. In addition, based on the dose-mortality relationship determined for each active substance, it was possible to assess the mortality induced at this exposure level (Figures S1, S2, S3, S4, S5, S6, S7, S8, S9, S10, S11, S12, S13, S14 and S15). For each active substance, two scenarios were presented: the most and the least protective that combined the highest LD_50_ and lowest field rate, for the most protective scenario, and the lowest LD_50_ and highest field rate, for the least protective one. The active substances were classified by their revisited HQ as a function of the different values of exposure calculated from the lower and the upper limits of the 95% confidence interval of the exposure surface area (Table S2). No differences in the classification of active substances were found when the lower and upper limits of the confidence interval were used to calculate the revisited HQ.

**Table 5:** Determination and comparison of the HQ and the revisited HQ.

Activesubstance	Time^a^(hours)	Application rate^b^(g a.s./ha)		LD_50_ c(µg a.s./bee)		HQ^d^		Exposure^e^(ng a.s./bee)		LD_50_ f(ng a.s./bee)		New HQ^g^(revisited)		Expectedmortality^h^	LD_50_References
Abamectin	24	Min	4.5	Max	0.002	Min	2045	Min	47	Max	2.2	Min	21.48	100%	[34]
	72	Max	22.5	Min	0.001	Max	15709	Max	236	Min	1.43	Max	164.94	100%	E.D. Figure S1
Acetamiprid	72	Min	5	Max	8.090	Min	0.6	Min	53	Max	8090	Min	0.006	2.2%	PED US EPA
	24	Max	100	Min	5.057	Max	20	Max	1050	Min	5057	Max	0.21	10.1%	E.D. Figure S2
Chlorpyrifos-ethyl	96	Min	187.5	Max	0.114	Min	1645	Min	1969	Max	114	Min	17.27	100%	PED US EPA
	96			E.D.	0.060					E.D.	59.52				E.D. Figure S3
	18	Max	1000	Min	0.010	Max	100000	Max	10500	Min	10	Max	1050	100%	PED US EPA
Clothianidin	48	Min	75	Max	0.044	Min	1695	Min	788	Max	44.26	Min	17.79	100%	Agritox Database
	24			E.D.	0.040					E.D.	40.23				E.D. Figure S4
	24	Max	75	Min	0.022	Max	3440	Max	788	Min	21.8	Max	36.12	100%	[35]
Cyfluthrin	48	Min	10	Max	0.037	Min	270	Min	105	Max	37	Min	2.84	99.1%	PED US EPA
	48			E.D.	0.020					E.D.	20.45				E.D. Figure S5
	48	Max	40	Min	0.001	Max	40000	Max	420	Min	1	Max	420	100%	[36]
Cypermethrin	24	Min	15	Max	0.121	Min	124	Min	158	Max	121.25	Min	1.30	71%	E.D. Figure S6
	24	Max	96	Min	0.020	Max	4800	Max	1008	Min	20	Max	50	99.9%	Agritox Database
Deltamethrin	24	Min	4.95	Max	0.108	Min	46	Min	52	Max	107.64	Min	0.48	7.4%	E.D. Figure S7
	48	Max	19.95	Min	0.002	Max	13300	Max	209	Min	1.5	Max	139.65	91.5%	PED US EPA
Dimethoate	24	Min	240	Max	0.454	Min	529	Min	2520	Max	454	Min	5.55	100%	[37]
	96			E.D.	0.163					E.D.	163.33				E.D. Figure S8
	48	Max	500	Min	0.001	Max	357143	Max	5250	Min	1.4	Max	3750	100%	[38]
Esfenvalerate	48	Min	1.5	Max	0.060	Min	25	Min	16	Max	60	Min	0.26	13.0%	[39]
	96			E.D.	0.047					E.D.	47.03				E.D. Figure S9
	48	Max	20	Min	0.017	Max	1163	Max	210	Min	17.2	Max	12.21	98.1%	PED US EPA
Fenoxycarb	48	Min	75	Max	>204	Min	<0.4	Min	788	Max	>204000	Min	<0.004	N.C.	[36]
	48	Max	150	Min	>100	Max	<2	Max	1575	Min	>100000	Max	<0.016	N.C.	[40]
Hexythiazox	48	Min	25	Max	>200	Min	<0.1	Min	263	Max	>200000	Min	<0.001	N.C.	[41]
	48	Max	50	Min	>200	Max	<0.3	Max	525	Min	>200000	Max	<0.003	N.C.	[41]
Imidacloprid	48	Min	50	Max	0.230	Min	217	Min	525	Max	230.3	Min	2.28	91.9%	[42]
	96			E.D.	0.096					E.D.	96.21				E.D. Figure S10
	48	Max	100	Min	0.007	Max	14925	Max	1050	Min	6.7	Max	156.72	96.8%	[43]
Iprodione	48	Min	75	Max	>200	Min	<0.4	Min	788	Max	>200000	Min	<0.004	N.C.	Agritox Database
	48	Max	1000	Min	>25	Max	<40	Max	10500	Min	>25000	Max	<0.42	N.C.	[36]
Lambda-cyhalothrin	48	Min	5	Max	0.098	Min	51	Min	53	Max	98	Min	0.54	23.2%	PED US EPA
	48			E.D.	0.078					E.D.	77.85				E.D. Figure S11
	48	Max	75	Min	0.038	Max	1974	Max	788	Min	38	Max	20.72	99.9%	PED US EPA
Prochloraz	96	Min	230.5	Max	141.28	Min	2	Min	2420	Max	141280	Min	0.02	40.7%	[44]
	24	Max	598.5	Min	2.657	Max	225	Max	6284	Min	2657	Max	2.37	97.5%	E.D. Figure S12
Pyriproxyfen	48	Min	25	Max	>100	Min	<0.3	Min	263	Max	>100000	Min	<0.003	N.C.	[45]
	48	Max	30	Min	74	Max	0.4	Max	315	Min	74000	Max	0.004	N.C.	PPDB IUPAC
Tau-fluvalinate	24	Min	48	Max	13	Min	4	Min	504	Max	12000	Min	0.04	2.1%	[46]
	48	Max	144	Min	2.532	Max	57	Max	1512	Min	2532	Max	0.60	17.4%	E.D. Figure S13
Tebuconazole	48	Min	75	Max	>200	Min	<0.4	Min	788	Max	>200000	Min	<0.004	N.C.	[47]
	48	Max	258	Min	>83	Max	<3.1	Max	2709	Min	>83050	Max	<0.033	N.C.	[36]
Thiacloprid	48	Min	72	Max	38.820	Min	2	Min	756	Max	38820	Min	0.02	4.1%	Agritox Database
	24			E.D.	19.380					E.D.	19380				E.D. Figure S14
	24	Max	180	Min	14.600	Max	12	Max	1890	Min	14600	Max	0.13	9.2%	[35]
Thiamethoxam	48	Min	50	Max	0.040	Min	1251	Min	525	Max	39.96	Min	13.14	100%	E.D. Figure S15
	24	Max	100	Min	0.024	Max	4167	Max	1050	Min	24	Max	44	100%	Agritox Database

g a.s./ha: mass of active substance per hectare expressed in grams.

µg a.s./bee: mass of active substance per bee expressed in micrograms.

ng a.s./bee: nanogram of active substance per bee.

E.D.: Experimental Data.

LD_50_: Median Lethal Dose.

HQ: Hazard Quotient (field rate (g/ha)/LD_50_ (µg/bee)).

Revisited HQ (exposure (ng/bee)/LD_50_ (µg/bee)).

N.C.: Not Calculated because of the low toxicity of the active substance.

DAR EFSA: Draft Assessment Report of the European Food Safety Authority.

PED US EPA: Pesticides Ecotoxicity Database of the United States Environmental Protection Agency.

a Time at which the LD_50_ was determined. The LD_50_ values resulting from the experimental data were calculated at the time corresponding to a stabilized mortality.

b For each active substance, 2 scenarios of exposure are presented: the lowest and the highest homologated application rate.

c For each active substance, the highest and the lowest known LD_50_ values were compared to the lowest and highest homologated application rates, respectively.

d HQ is the ratio between the application rate (g a.s./ha) and the LD_50_ (µg a.s./bee).

e The exposure was calculated from the application rate (ng a.s./cm^2^) and the mean exposure surface area determined with the 20 active substances (1.05 cm^2^/bee).

f The LD_50_ values from the experimental data were calculated with the BMD software from the US EPA.

g The revisited HQ is the ratio between the exposure (ng a.s./bee) and the LD_50_ (ng a.s./bee).

h For each active substance, the dose-mortality relationship was modeled at the time corresponding to a stabilized mortality.

### Determination of the physical surface area of a bee

**Figure 3:**
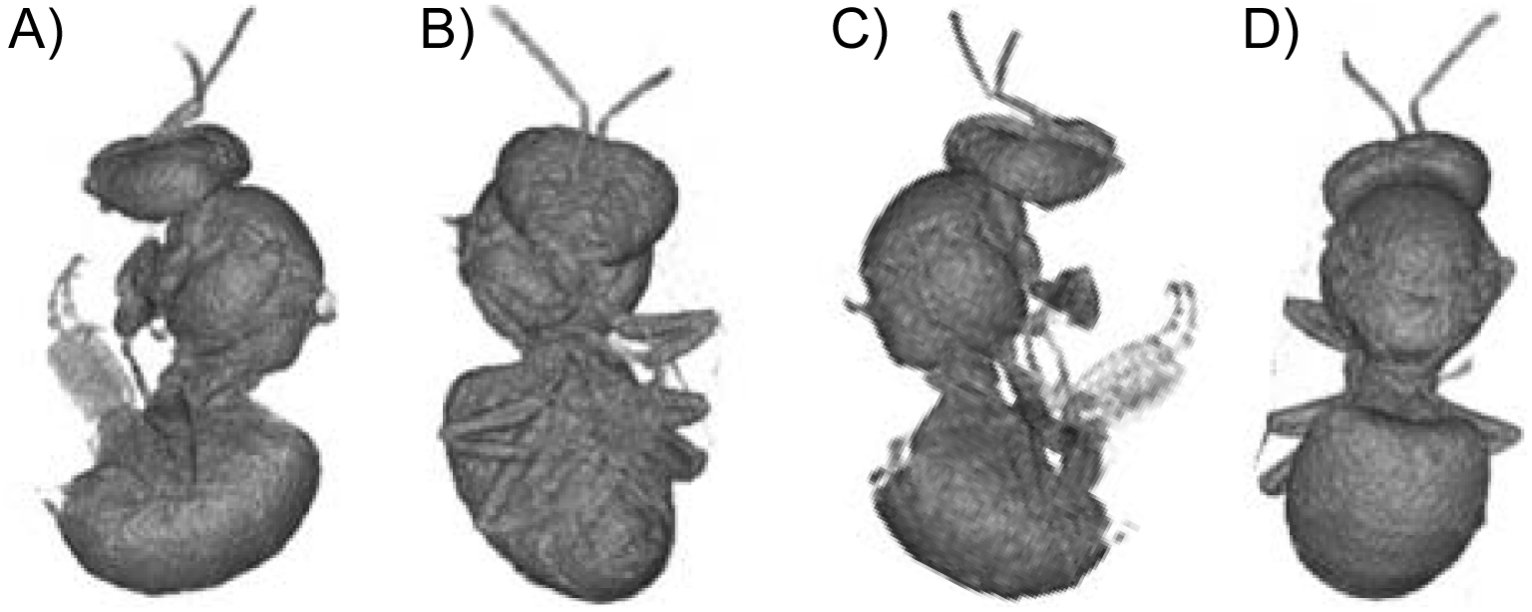
3D representation of a bee from different angles. Bees were scanned using X-ray tomography. A: Left lateral view; B: Ventral view; C: Right lateral view; D: Dorsal view.

**Figure 4:**
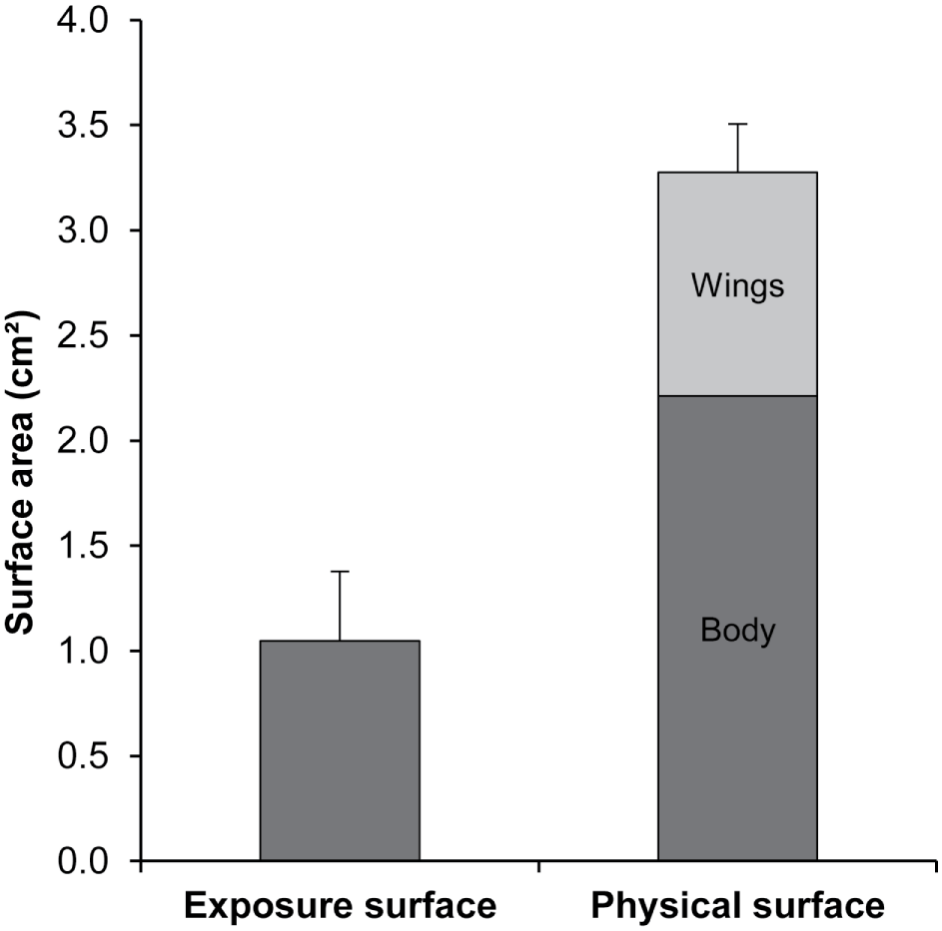
Comparison of the exposure and physical surface area of a bee. Mean exposure surface area (± S.D.) of a bee (average of the 20 commercial products) and mean physical surface area (± S.D.) of a bee (sum of the mean body and wing surface area).

To investigate the possibility of an overestimation of the exposure surface area of bees, the physical surface area of a bee was determined by X-Ray tomography. The surface areas of the body and the wings were measured separately. The tomographic analyses resulted in 3-D representation of the bees (Figure 3) that enabled calculating the volume (not relevant here) and the surface area of a bee. The exposure surface area was compared to the physical surface area of a bee (Figure 4). The mean body surface area was 2.21±0.20 cm^2^ (n = 6), the mean wing surface area was 1.06±0.03 cm^2^ (n = 50) and the mean total physical surface area of a bee was 3.27±0.23 cm^2^. It was noteworthy that the wings represented almost one third of the total physical surface area of a bee and that the physical surface area was 3 times higher than the apparent exposure surface area.

## Discussion

In this study, we used a pragmatic approach to determine the exposure level of a bee submitted to spraying by an agrochemical at a known field rate. This exposure level enabled assessing the exposure surface area of a honey bee that could be used in the assessment of the risk posed by plant protection products as recommended by the EFSA [11]. The mean apparent exposure surface area was estimated as 1.05±0.33 cm^2^/bee (n = 20). Various attempts have been made to estimate the apparent exposure surface area of a bee. The first involves the determination of the apparent surface area by projection from photographs, which results in a surface area of approximately 0.5 cm^2^/bee [23]. We used this method in a previous study to transform field rates into doses received by bees with a good correlation between mortality rates [24]. The second method is more pragmatic and has been performed in an original study by Koch and Weisser, in which caged bees were submitted to a spraying of fluorescein at a field rate of 20 g/ha [25]. With this procedure, exposure levels ranged from 1.62 to 8.51 ng/bee in apple orchards and from 6.34 to 35.77 ng/bee in phacelia. These values correspond to an apparent exposure surface area ranging from 0.0081 to 0.0425 cm^2^/bee in apple orchards and 0.0317 to 0.1789 cm^2^/bee in phacelia, which is very different from the surface area determined in the present study. Two phenomena can explain the great discrepancy between these exposure values and those presented here. (i) The fluorescein recovery was achieved by rinsing the bees, which does not enable the extraction of the absorbed fluorescein and leads to important underestimation of fluorescein residues. In our approach, pesticide residues were recovered by an efficient extraction procedure that presents high recovery rates [26]. (ii) In our study, bees were immediately frozen after spraying, which stops the biotransformation of the pesticides. However, in the study of Koch and Weisser, the time elapsed between field exposure and sampling was much higher and close to 20 min. In addition, during the sampling the bees were cooled with dry ice and subsequently frozen in the laboratory. Thus, fluorescein was subjected to biotransformation during the sampling procedure, which can be very rapid; this has been previously observed with pesticides for which *in* *vivo* half-lives as low as 25 min have been observed [27]. It is noteworthy that the effect of time on fluorescein recovery was described by Koch and Weisser.

For a better assessment, the apparent exposure surface area was estimated with 20 pesticides from several chemical groups. Slight variations in the surface area have been observed from pesticide to pesticide. However, the concentrations of the pesticide solutions were checked analytically, the recovery rates of pesticide extraction were very high and we made sure that the deposit was very constant over time (Table 4). Thus, variations of the apparent exposure surface area were not due to variations in the quantity of the deposited pesticide solution but due to the quantities of the deposited active substance, which depends on their physico-chemical properties and on the components of the pesticide formulations [28].

To test the accuracy of the estimated exposure surface area, the toxicity elicited by sprayings at given field rates was compared with the toxicity induced by the corresponding doses calculated on the basis of the field rates and the exposure surface area. The linear regression analysis showed not only a very good correlation (*r^2^* = 0.960) between mortality induced by field doses and that induced by doses calculated with the apparent exposure surface area but also a slope of 0.9792, which is very close to 1. A slope higher or lower than 1 would have revealed an overestimation or underestimation of the apparent exposure surface area, respectively. Thus, a slope of approximately 1 clearly shows that the toxicity induced by spraying at a given field rate can be predicted by the toxicity induced by contact treatment according to the EPPO procedure with the corresponding dose calculated on the basis of the field rate and the apparent exposure surface area of a bee. In other words, this shows that the apparent exposure surface area of a bee can be used in procedures to assess the risk presented by pesticides to bees. Hence, it is possible to derive two formulas that could be used in risk assessment. The first enables calculating the individual dose received by the bees (*ID*) during the spraying of a pesticide at a given field rate:
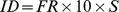
where *ID* is the individual dose expressed in ng/bee; *FR* is the field rate of the pesticide expressed in g/ha and *S* is the apparent exposure surface area of 1.05 cm^2^/bee. The factor 10 results from the conversion of *FR* in g/ha into *FR* in ng/cm^2^. It is noteworthy that the standard deviation (0.33 cm^2^/bee) or the upper limit of the confidence interval (1.21 cm^2^/bee) offers the possibility to consider worse case scenarios of exposure during spraying. The second formula can be used to estimate the field rate that should not be exceeded when a reference value, such as LD_50_ or acute reference dose (ARfD), is available to characterize the toxicity of a pesticide:



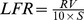
where *LFR* is the limit field rate that should not be exceeded expressed in g/ha, *RV* is the reference value expressed in ng/bee and *S* is the apparent exposure surface area of 1.05 cm^2^/bee.

In this study, we propose a new approach for the determination of the hazard quotient (HQ) in the procedure used for the assessment of the risk presented by pesticides to the honey bee. The HQ is no longer estimated from the field application rates of pesticides (in g/ha) but instead from the exposure to which the bees are subjected (in ng/bee). Thus, the resulting HQ is a value without units because it corresponds to the ratio of coherent exposure and toxicity data. The environmental exposure level to which bees are subjected is expressed in ng/bee. The toxicity, corresponding to the exposure level from which effects may be considered damaging to the bees (or to a bee colony), is also expressed in ng/bee. This approach is used for all living organisms in risk assessment procedures, including humans [3], [29]. Until now, the honey bee remained the only organism for which the HQ value was interpreted empirically to manage the risk to bees because it was not based on the ratio of comparable data. This point has also been raised by one agency of the European Union, the DG SANCO [30]. The determination of the exposure surface area of a bee also enables converting previous HQ values into new HQ values (revisited HQ) (*new HQ = 1.05*×*10^−2^*×*HQ*), which in turn enables using the old toxicological data. Moreover, the apparent exposure surface area enables the assessment of the survival probability of bees sprayed by a pesticide at a given field rate on the basis of the dose-mortality relationship. Thus, the combination of the new HQ with the expected mortality and exposure could be useful tools for decision-making processes in the assessment of pesticide toxicity to the honey bee. However, although the revisited HQ gives a better interpretation of the potential risk presented by a pesticide than the previous HQ, the exposure is still compared to a toxicological value that elicits a mortality rate of 50% in the honey bee risk assessment procedure. For humans and some target organisms (i.e., fish), and for specific toxicity, such as reproductive toxicity, for birds and mammals [31], the risk is based on toxic reference values at which a low mortality or no effect is observed (e.g., NOAEL, NOAEC, ARfD, or Benchmark dose). Consequently, it appears legitimate to reconsider the LD_50_ value as a reference value to assess the risk presented by pesticides to bees.

The apparent exposure surface area of 1.05 cm^2^/bee does not appear overestimated compared with the physical surface area of 3.27 cm^2^/bee. The physical surface area measured in the present study appears to be higher than that determined in previous studies using graph paper [32], [33]. Although X-Ray tomography is not yet sufficiently powerful to take into account the surface hairs on the honeybee thorax, this technique remains more precise than the others described previously. The difference between exposure and physical surface areas can be explained by the fact that the entire surface was not available during the spraying. Indeed, the bees were motionless with their wings folded on the body during the spraying. Then, the exposure surface area does not depend on the total surface area (body + wings = 3.27 cm^2^), but is rather a reflection of the body surface area (2.21 cm^2^). In this study, the bees were subjected to pesticides on one side, like during a field spraying. Thus, only one half of the body was directly exposed (c.a. 1.11 cm^2^), which is close to the apparent exposure surface area (1.05 cm^2^).

## Supporting Information

Figure S1 **Dose-mortality relationship of honey bees after a single contact contamination of abamectin on the thorax.**
(TIF)

Figure S2 **Dose-mortality relationship of honey bees after a single contact contamination of acetamiprid on the thorax.**
(TIF)

Figure S3 **Dose-mortality relationship of honey bees after a single contact contamination of chlorpyrifos-ethyl on the thorax.**
(TIF)

Figure S4 **Dose-mortality relationship of honey bees after a single contact contamination of clothianidin on the thorax.**
(TIF)

Figure S5 **Dose-mortality relationship of honey bees after a single contact contamination of cyfluthrin on the thorax.**
(TIF)

Figure S6 **Dose-mortality relationship of honey bees after a single contact contamination of cypermethrin on the thorax.**
(TIF)

Figure S7 **Dose-mortality relationship of honey bees after a single contact contamination of deltamethrin on the thorax.**
(TIF)

Figure S8 **Dose-mortality relationship of honey bees after a single contact contamination of dimethoate on the thorax.**
(TIF)

Figure S9 **Dose-mortality relationship of honey bees after a single contact contamination of esfenvalerate on the thorax.**
(TIF)

Figure S10 **Dose-mortality relationship of honey bees after a single contact contamination of imidacloprid on the thorax.**
(TIF)

Figure S11 **Dose-mortality relationship of honey bees after a single contact contamination of lambda-cyhalothrin on the thorax.**
(TIF)

Figure S12 **Dose-mortality relationship of honey bees after a single contact contamination of prochloraz on the thorax.**
(TIF)

Figure S13 **Dose-mortality relationship of honey bees after a single contact contamination of tau-fluvalinate on the thorax.**
(TIF)

Figure S14 **Dose-mortality relationship of honey bees after a single contact contamination of thiacloprid on the thorax.**
(TIF)

Figure S15 **Dose-mortality relationship of honey bees after a single contact contamination of thiamethoxam on the thorax.**
(TIF)

Table S1 **Active substances and their doses used for exposures through thoracic topical exposure.** ng a.s./bee: nanogram of active substance per bee. Nr: Number of replicates per dose. ABA = Abamectin, ACE = Acetamiprid, CHL = Chlorpyrifos-ethyl, CLO = Clothianidin, CYF = Cyfluthrin, CYP = Cypermethrin, DEL = Deltamethrin, DIM = Dimethoate, ESF = Esfenvalerate, IMI = Imidacloprid, LAM = Lambda-cyhalothin, PRO = Prochoraz, TAU = Tau-fluvalinate, THI = Thiacloprid, TMX = Thiamethoxam.
(DOCX)

Table S2 **Classification of the 20 active substances by their revisited HQs for different values of exposure surface areas.** An active substance was classified with a HQ≥1 if at least the revisited HQ of one of the two scenarios (Table 5) were equal to or higher than this value. Lower C.I.: Lower limit of the Confidence Interval at 95%. Upper C.I.: Upper limit of the Confidence Interval at 95%.
(DOCX)
