# Supplementary figures and images for: A Pragmatic Approach to Assess the Exposure of the Honey Bee (Apis mellifera) When Subjected to Pesticide Spray
Source: PLoS One. 2014 Nov 20;9(11):e113728. doi: 10.1371/journal.pone.0113728 (PMC4239102; doi:10.1371/journal.pone.0113728)

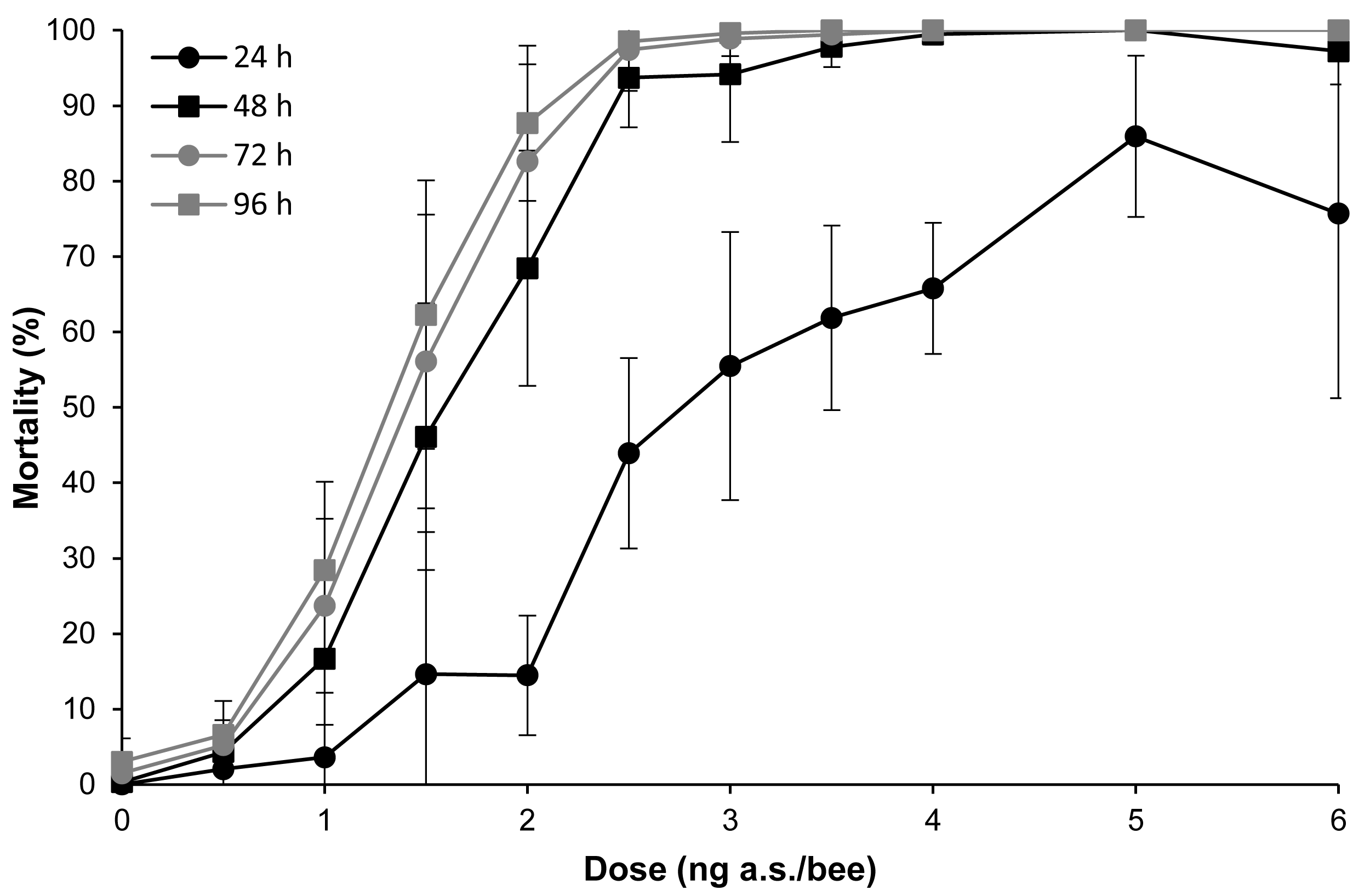

Supplement: Figure S1 — Dose-mortality relationship of honey bees after a single contact contamination of abamectin on the thorax. (TIF) [file pone.0113728.s001.tif]

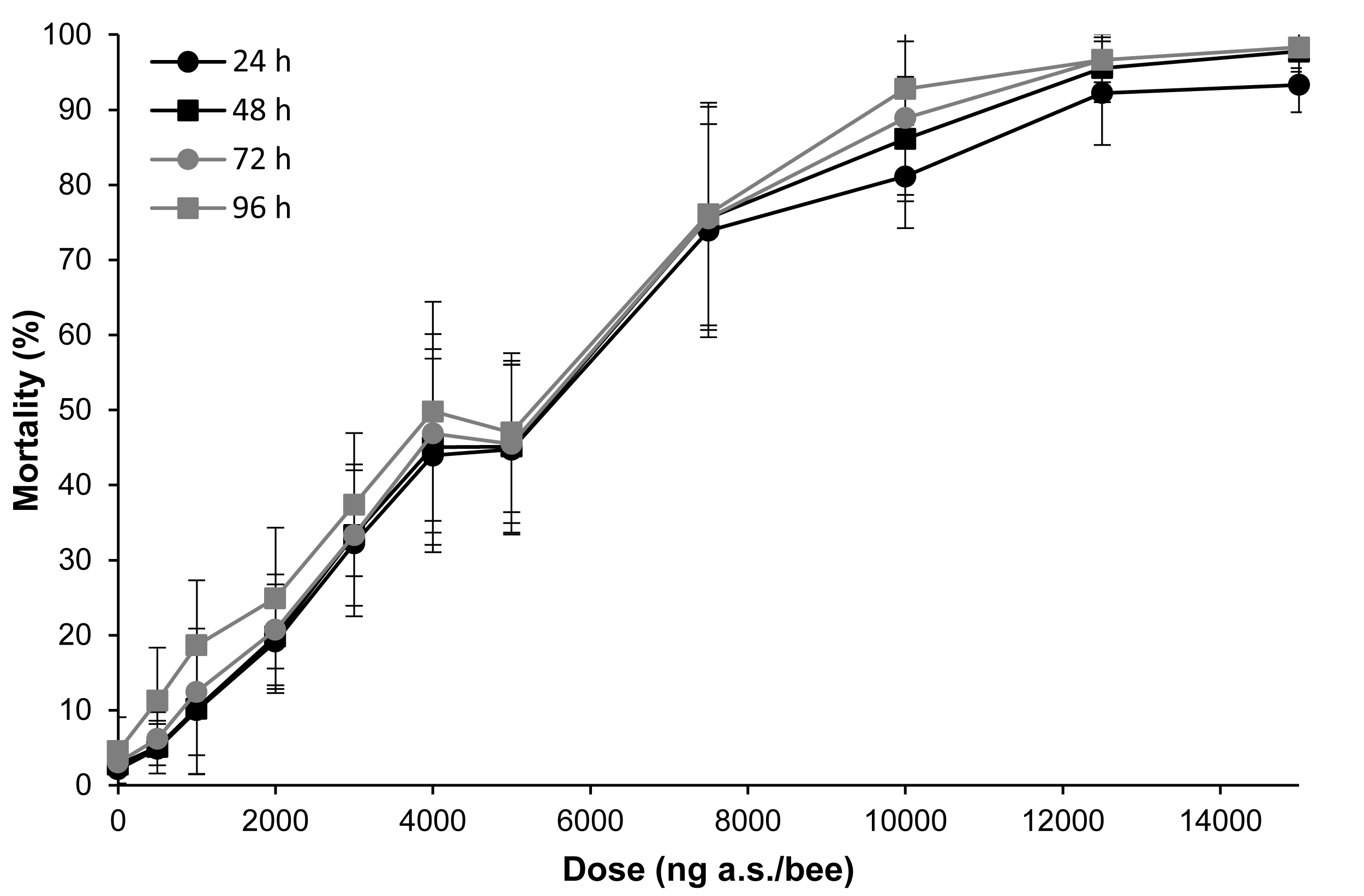

Supplement: Figure S2 — Dose-mortality relationship of honey bees after a single contact contamination of acetamiprid on the thorax. (TIF) [file pone.0113728.s002.tif]

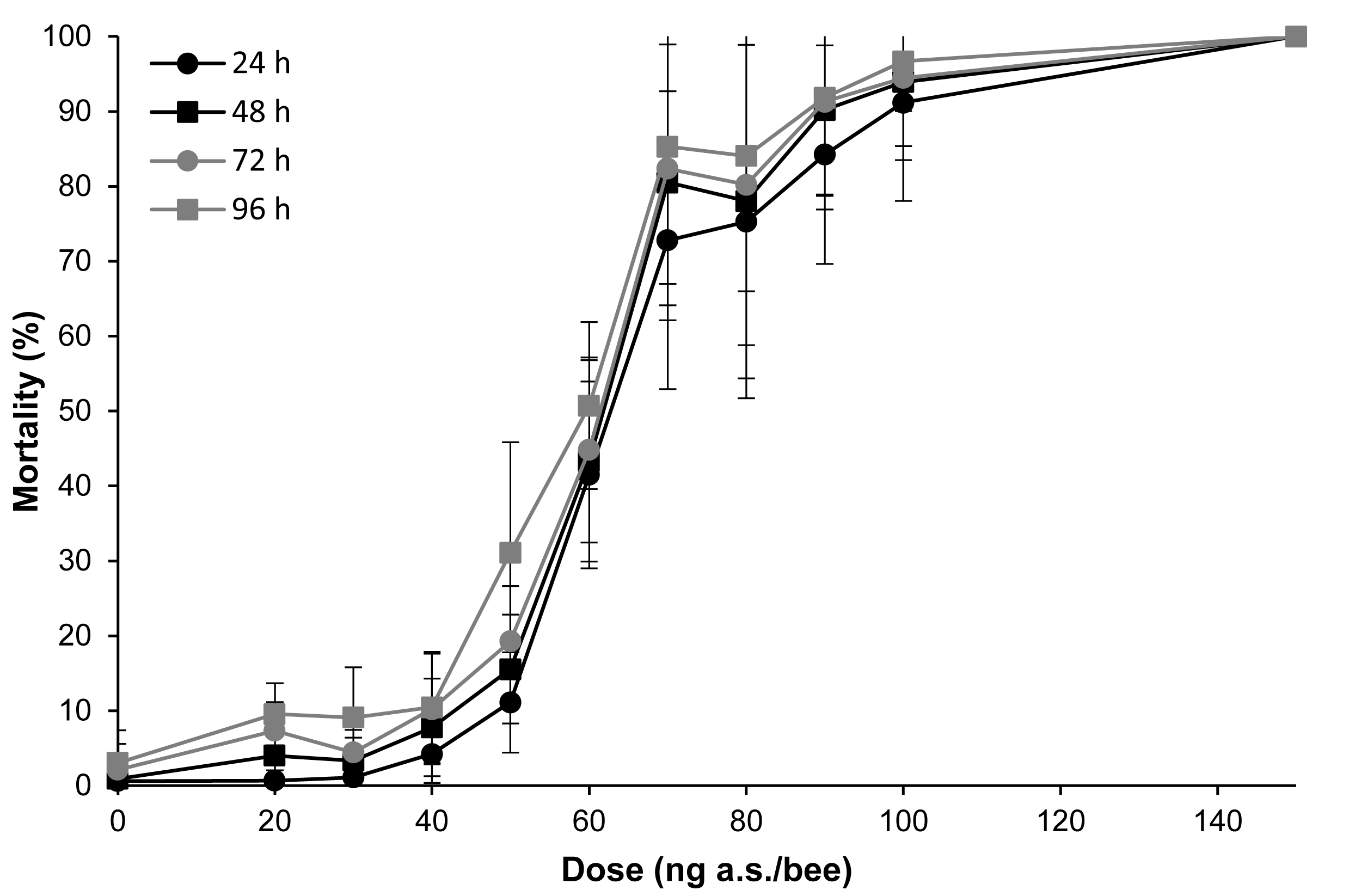

Supplement: Figure S3 — Dose-mortality relationship of honey bees after a single contact contamination of chlorpyrifos-ethyl on the thorax. (TIF) [file pone.0113728.s003.tif]

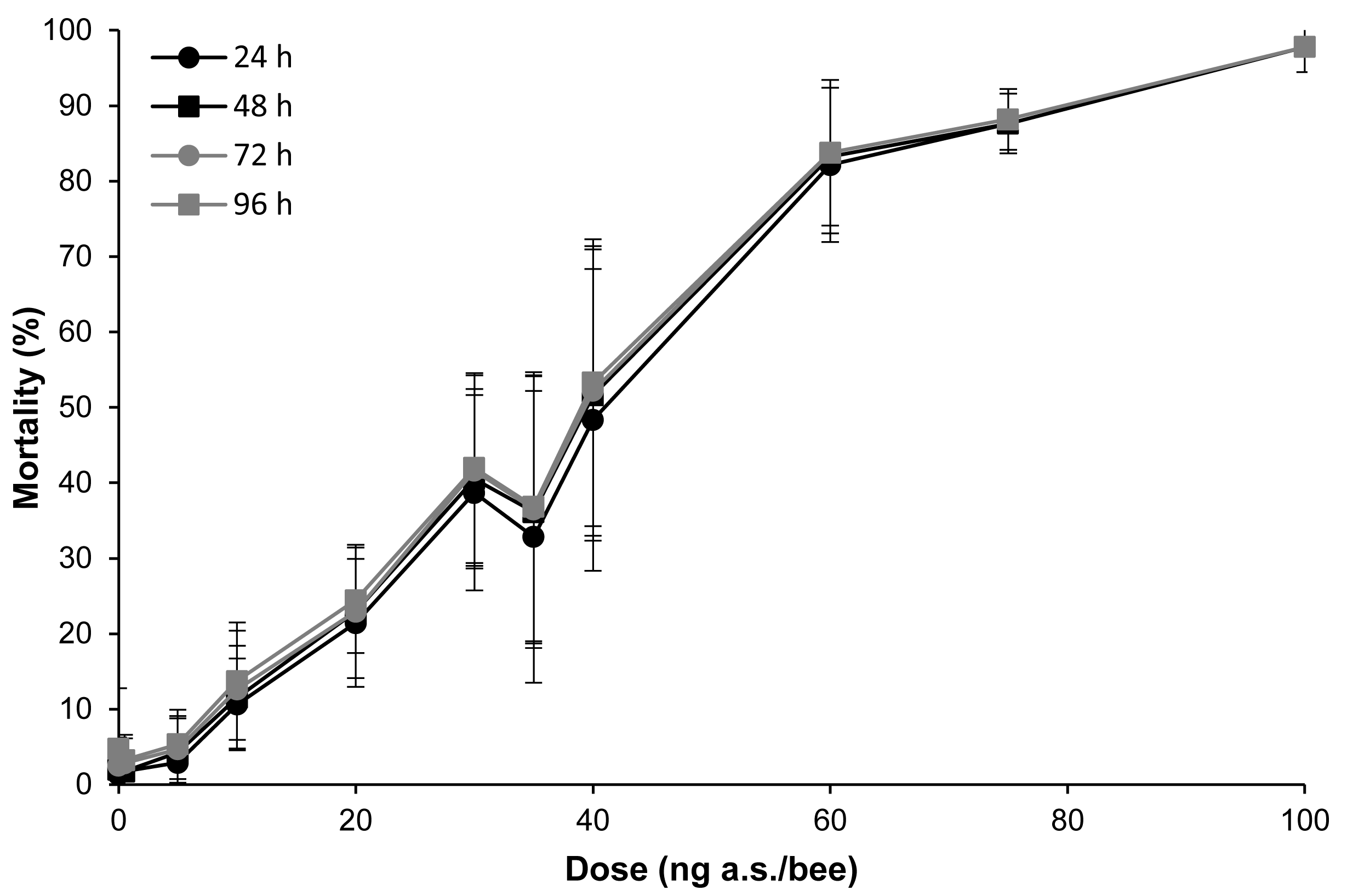

Supplement: Figure S4 — Dose-mortality relationship of honey bees after a single contact contamination of clothianidin on the thorax. (TIF) [file pone.0113728.s004.tif]

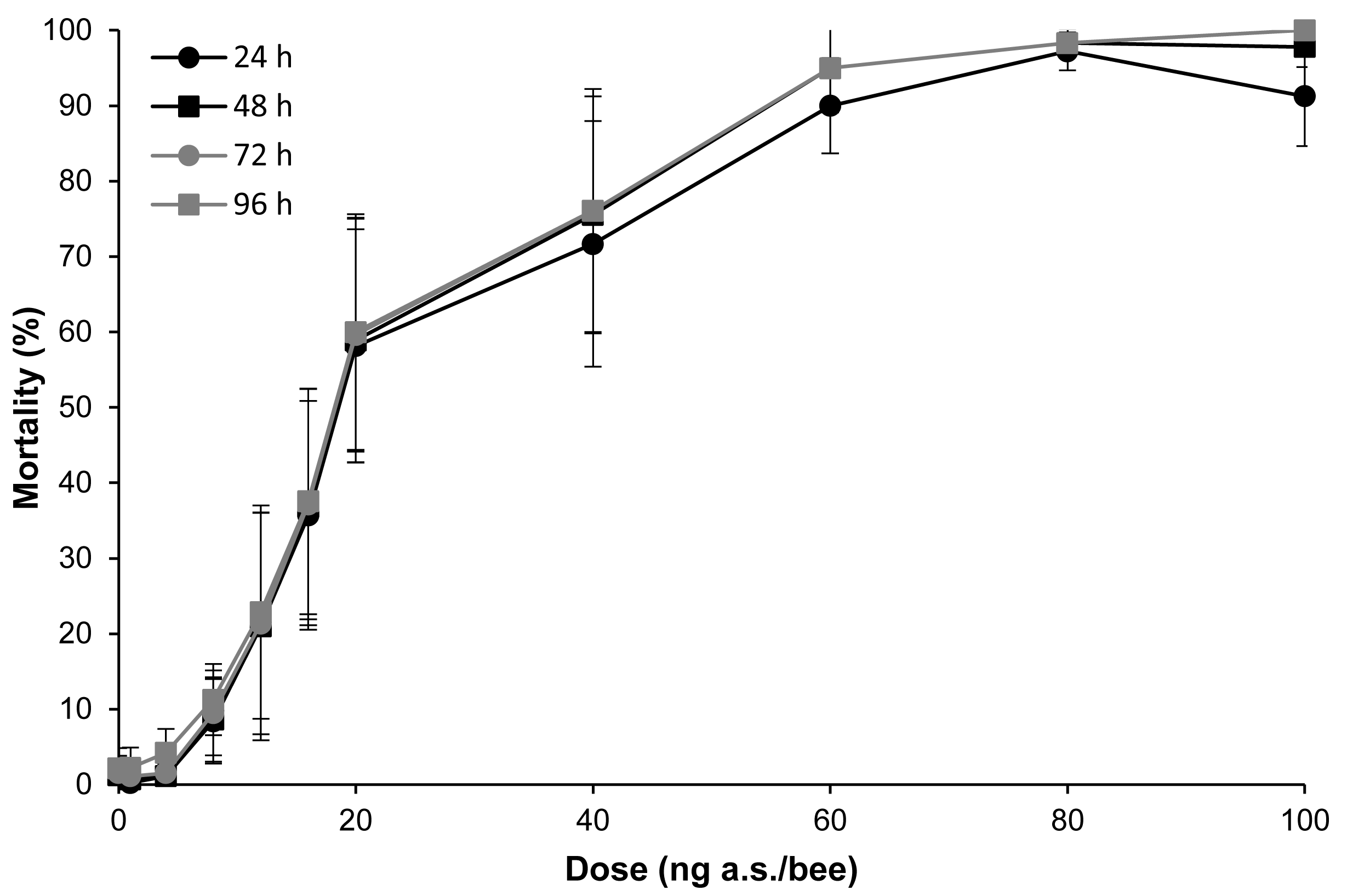

Supplement: Figure S5 — Dose-mortality relationship of honey bees after a single contact contamination of cyfluthrin on the thorax. (TIF) [file pone.0113728.s005.tif]

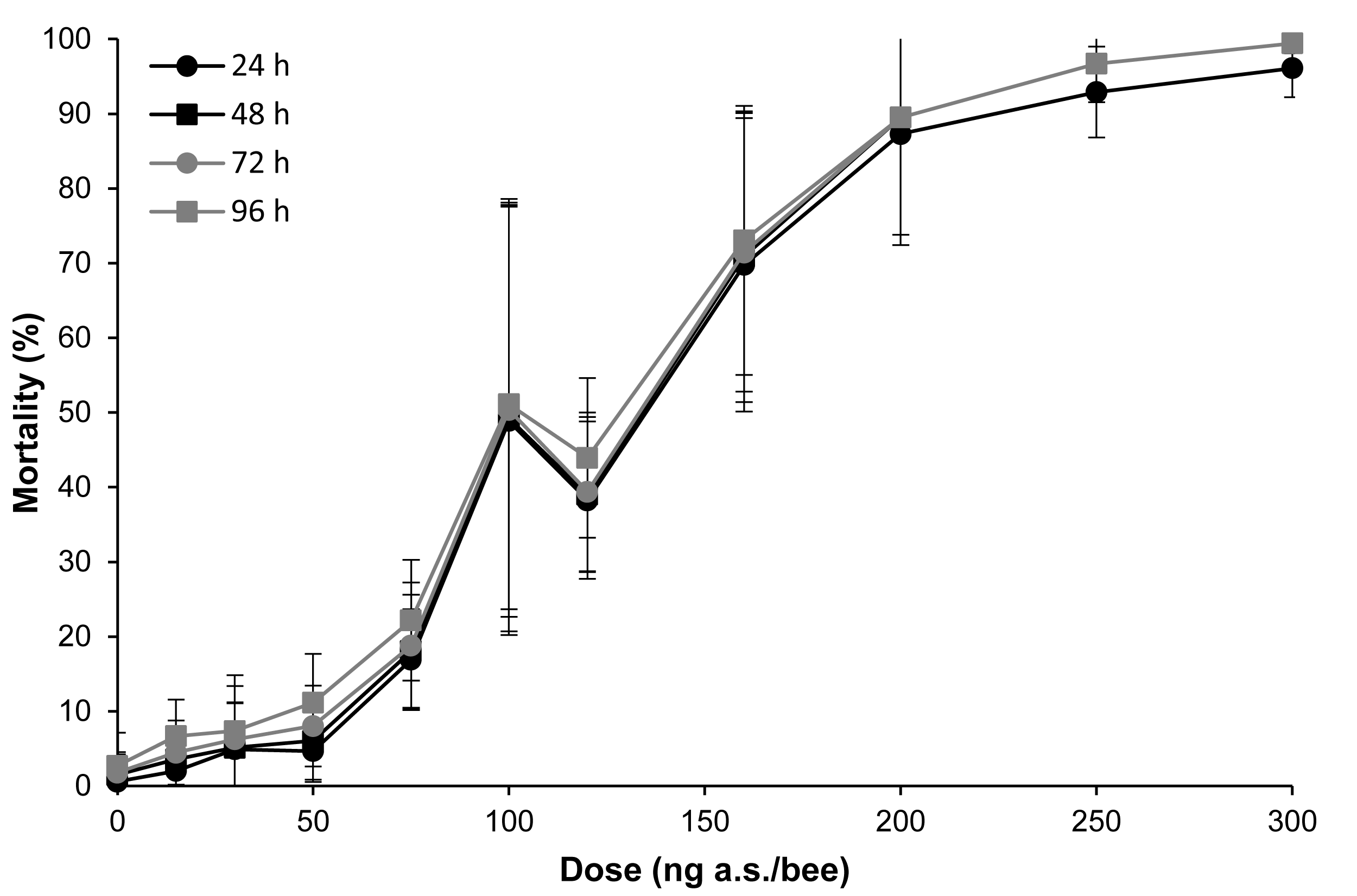

Supplement: Figure S6 — Dose-mortality relationship of honey bees after a single contact contamination of cypermethrin on the thorax. (TIF) [file pone.0113728.s006.tif]

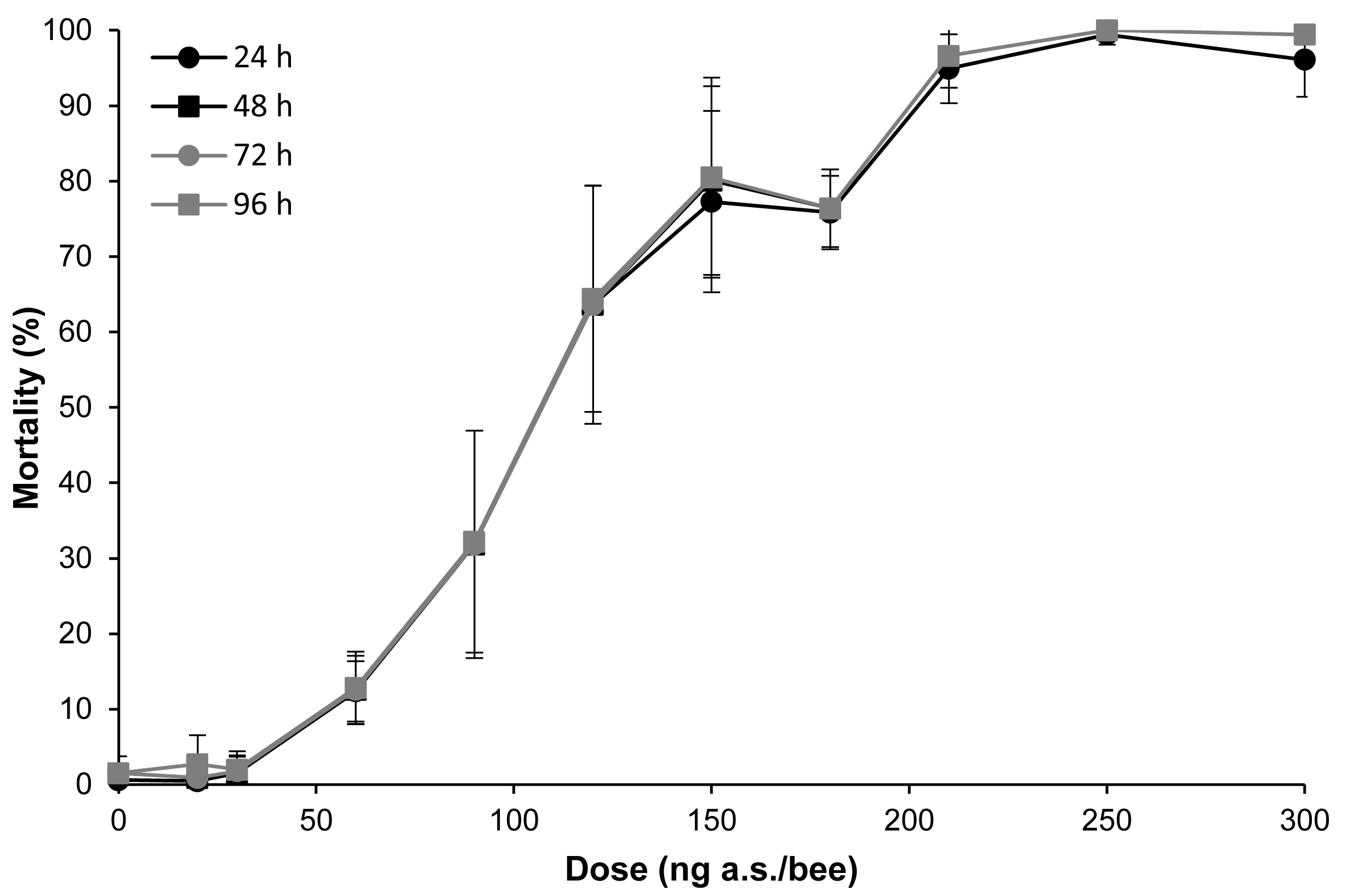

Supplement: Figure S7 — Dose-mortality relationship of honey bees after a single contact contamination of deltamethrin on the thorax. (TIF) [file pone.0113728.s007.tif]

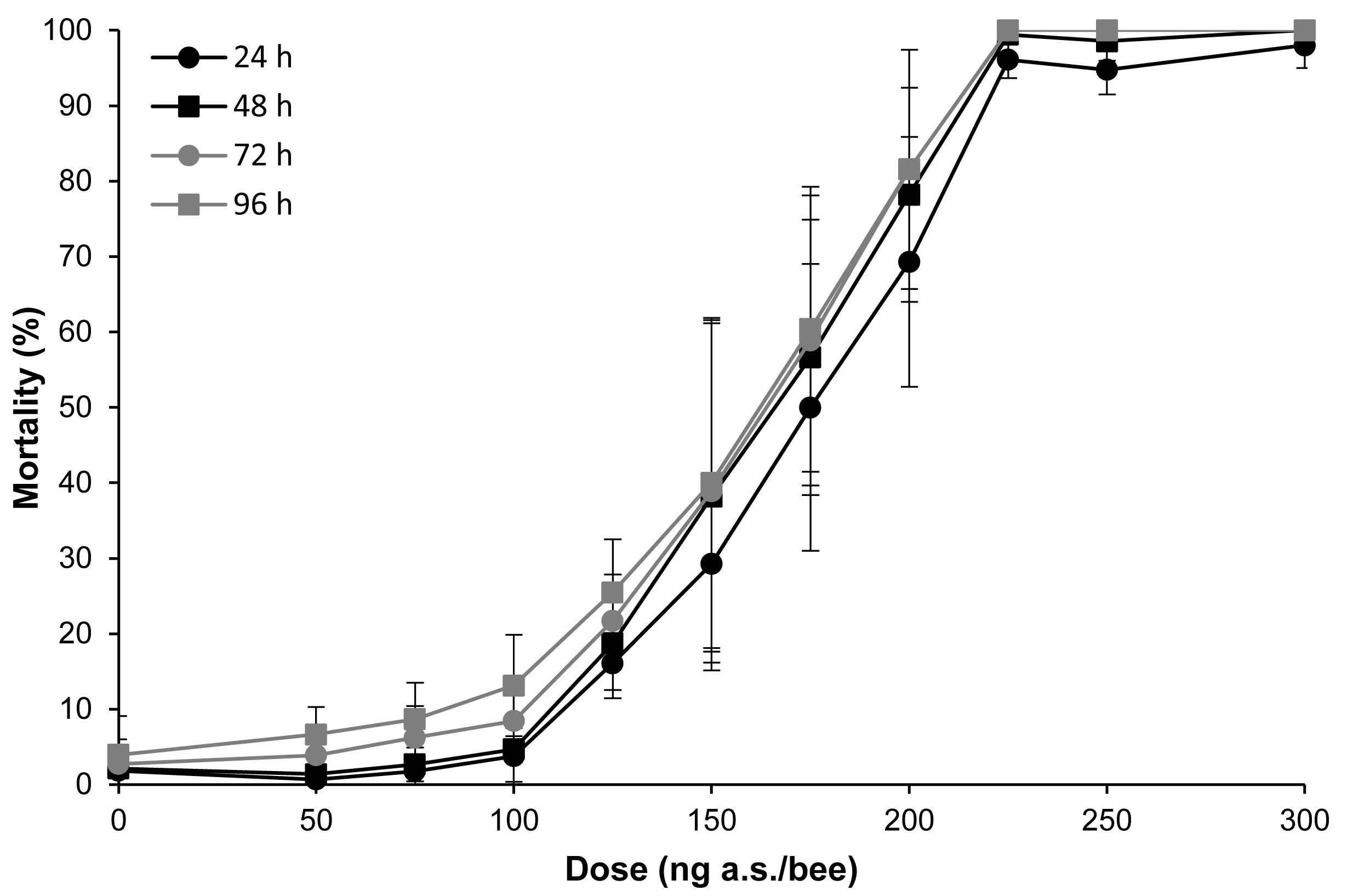

Supplement: Figure S8 — Dose-mortality relationship of honey bees after a single contact contamination of dimethoate on the thorax. (TIF) [file pone.0113728.s008.tif]

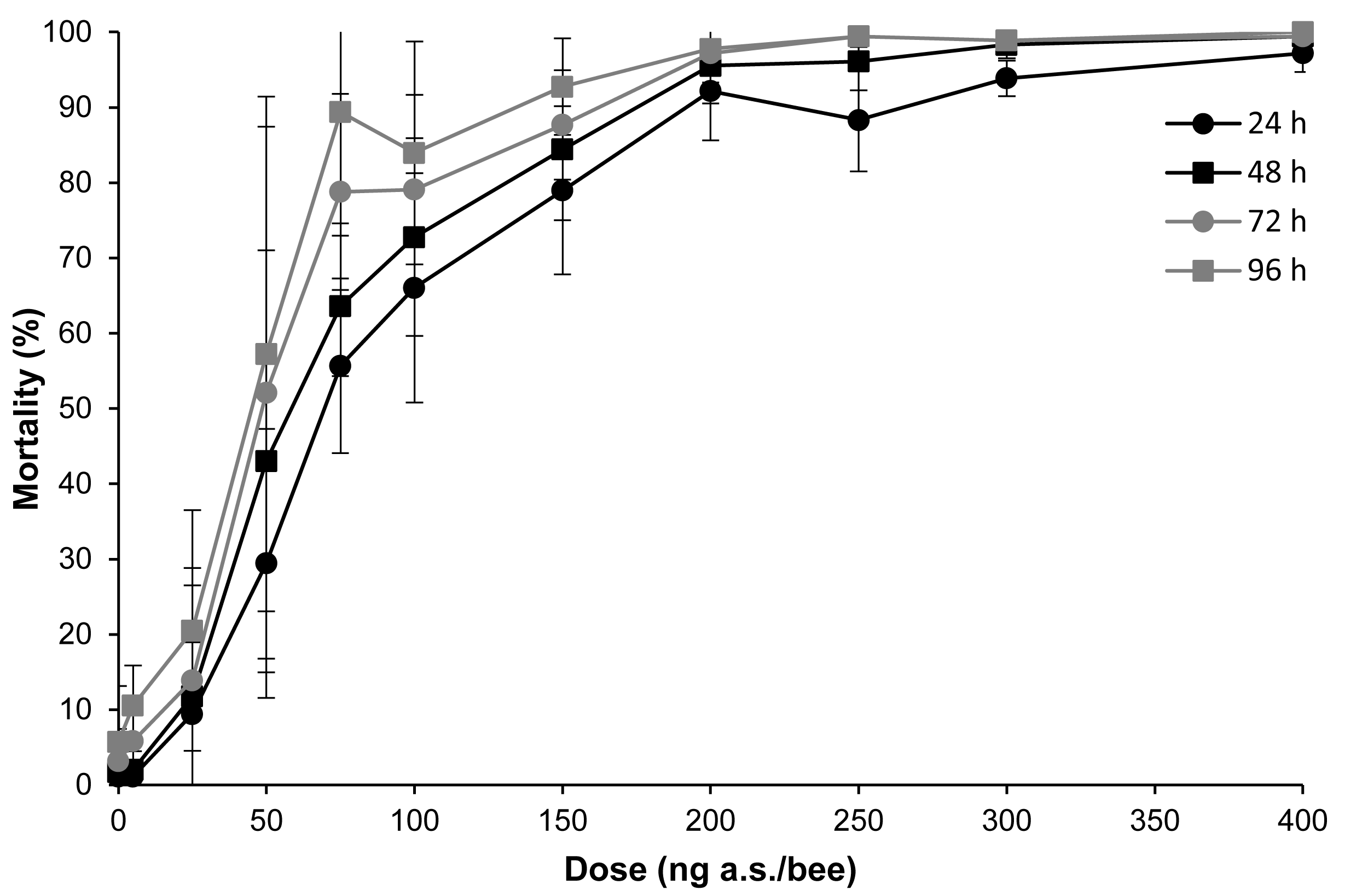

Supplement: Figure S9 — Dose-mortality relationship of honey bees after a single contact contamination of esfenvalerate on the thorax. (TIF) [file pone.0113728.s009.tif]

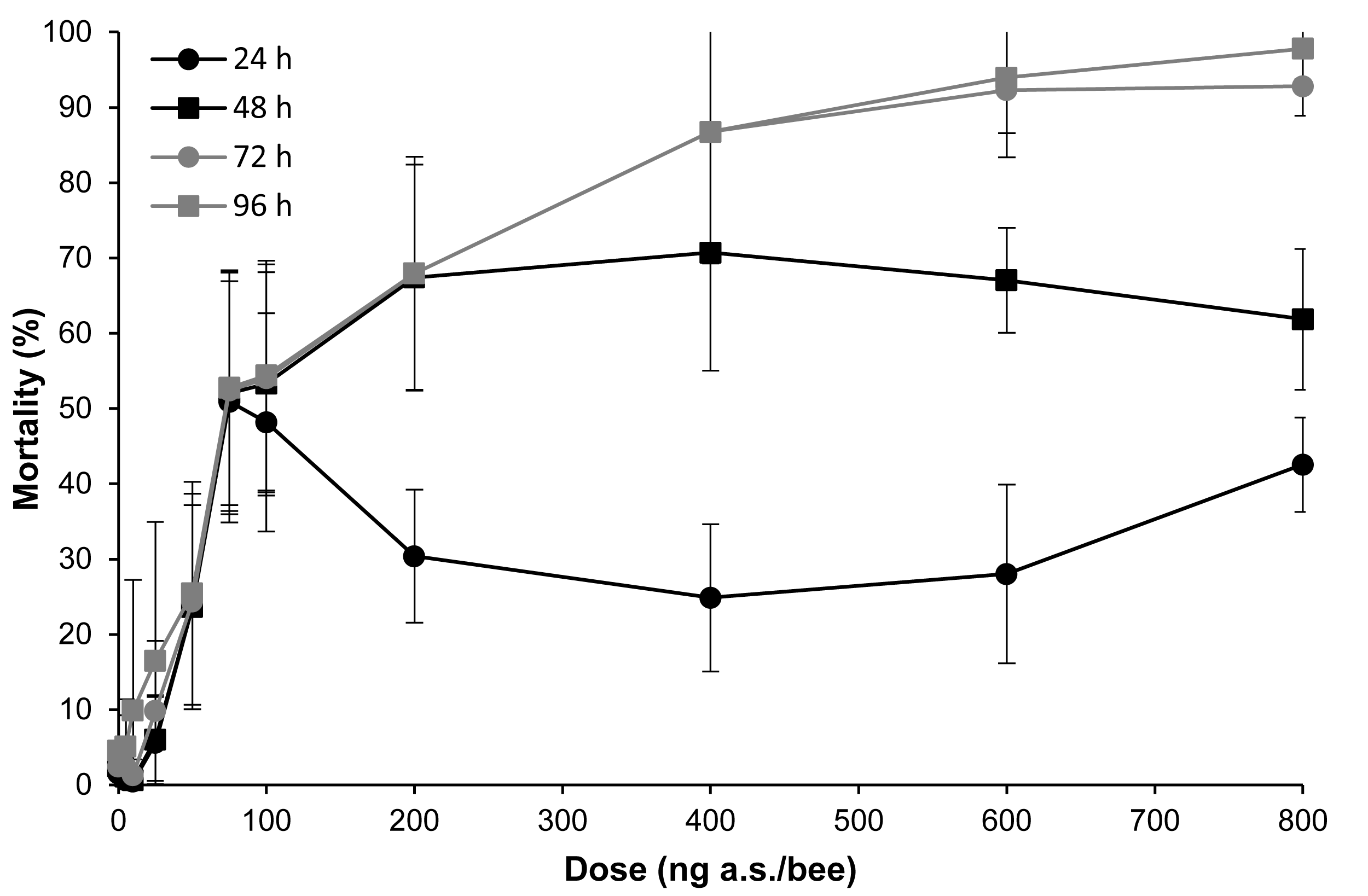

Supplement: Figure S10 — Dose-mortality relationship of honey bees after a single contact contamination of imidacloprid on the thorax. (TIF) [file pone.0113728.s010.tif]

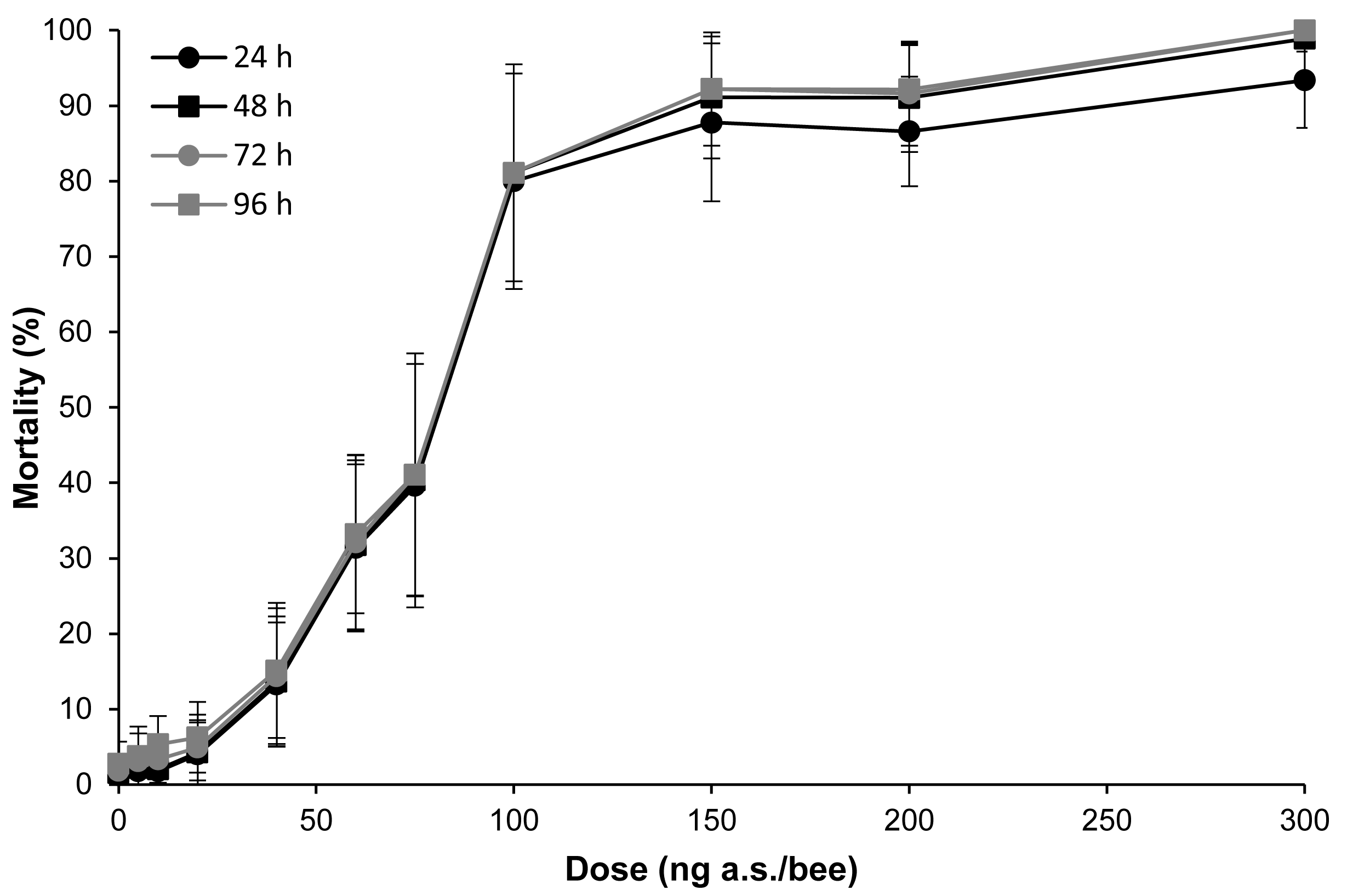

Supplement: Figure S11 — Dose-mortality relationship of honey bees after a single contact contamination of lambda-cyhalothrin on the thorax. (TIF) [file pone.0113728.s011.tif]

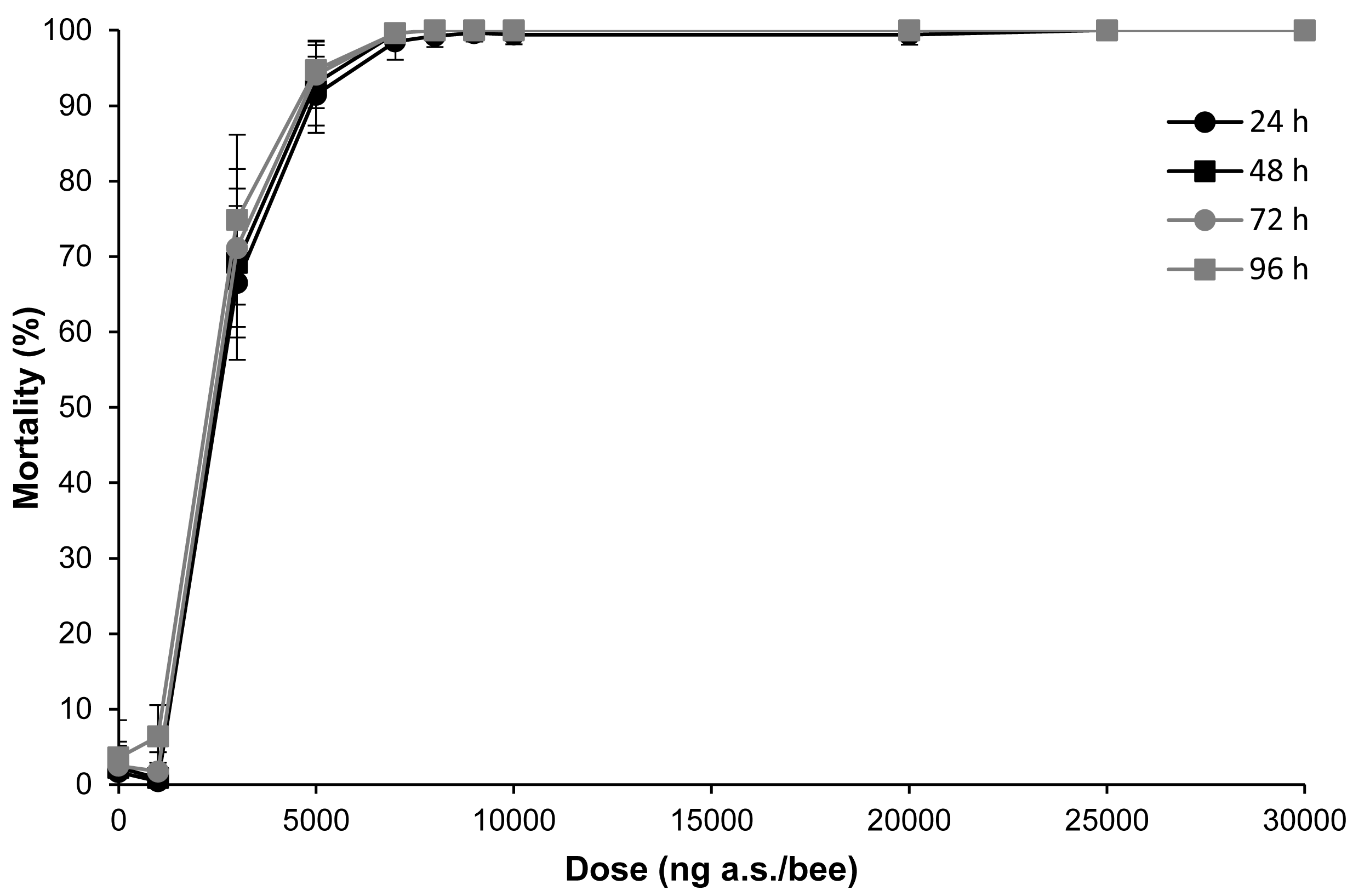

Supplement: Figure S12 — Dose-mortality relationship of honey bees after a single contact contamination of prochloraz on the thorax. (TIF) [file pone.0113728.s012.tif]

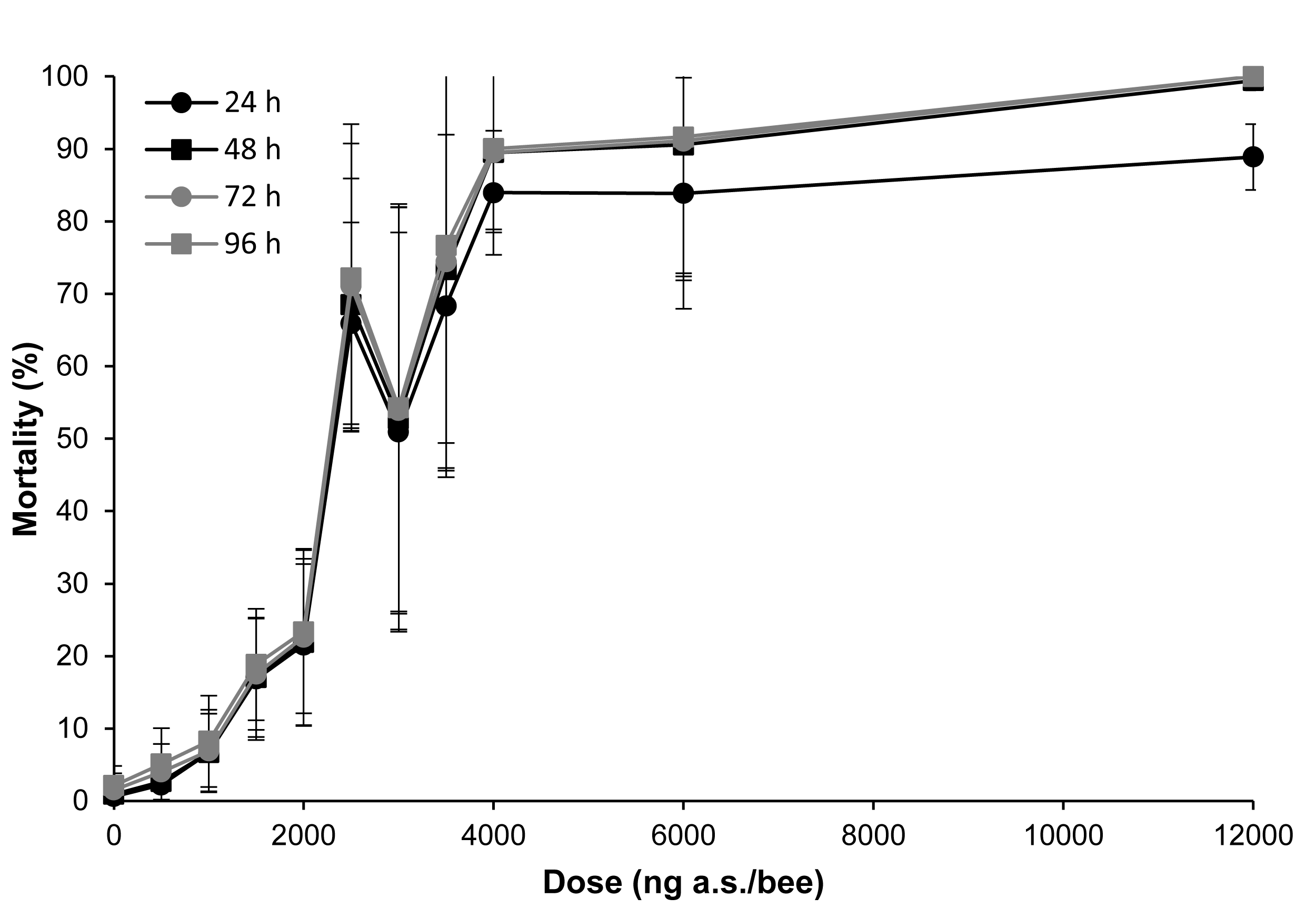

Supplement: Figure S13 — Dose-mortality relationship of honey bees after a single contact contamination of tau-fluvalinate on the thorax. (TIF) [file pone.0113728.s013.tif]

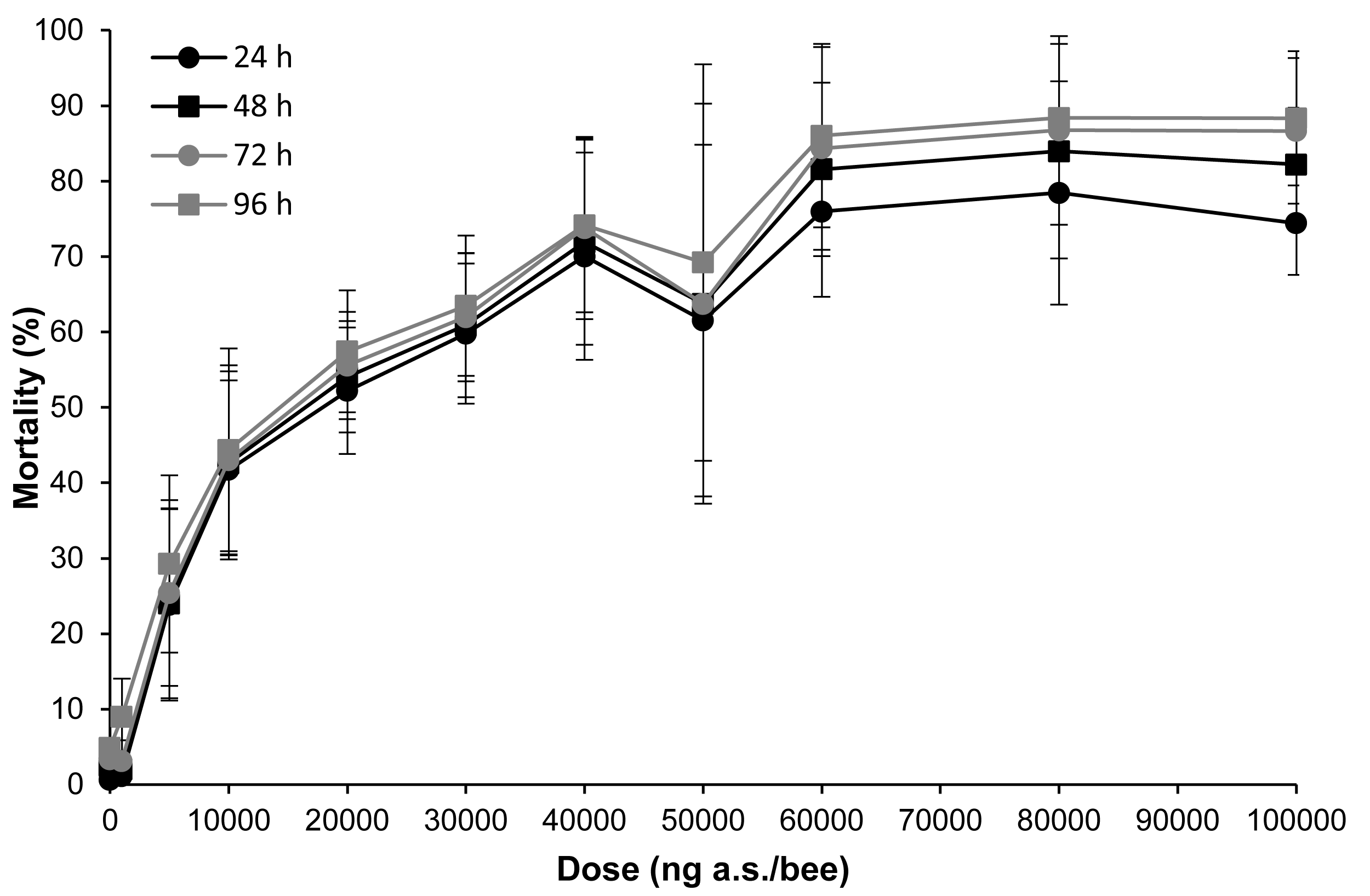

Supplement: Figure S14 — Dose-mortality relationship of honey bees after a single contact contamination of thiacloprid on the thorax. (TIF) [file pone.0113728.s014.tif]

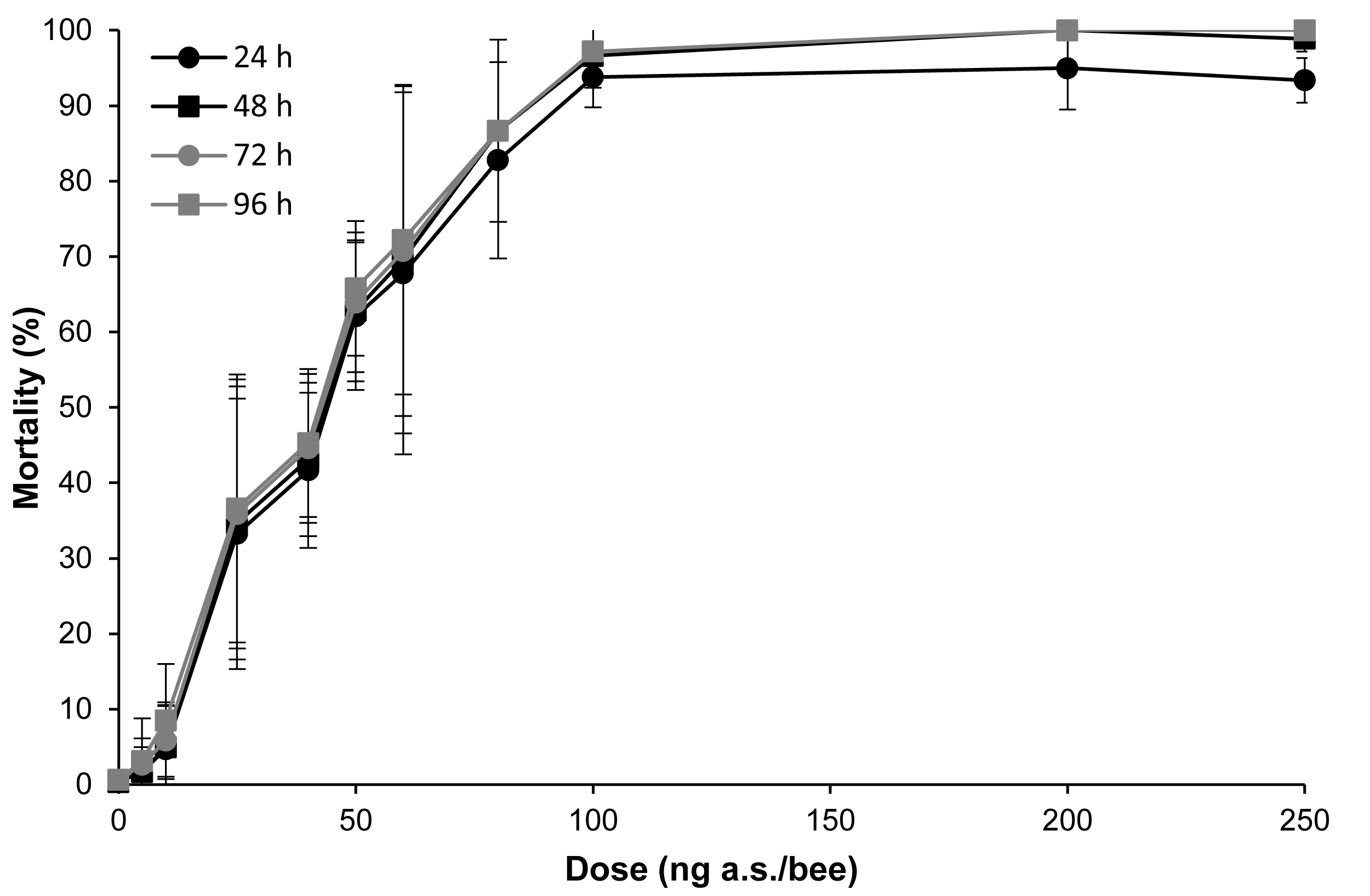

Supplement: Figure S15 — Dose-mortality relationship of honey bees after a single contact contamination of thiamethoxam on the thorax. (TIF) [file pone.0113728.s015.tif]
