# Supplementary material for: A Pragmatic Approach to Assess the Exposure of the Honey Bee (Apis mellifera) When Subjected to Pesticide Spray
Source: PLoS One. 2014 Nov 20;9(11):e113728. doi: 10.1371/journal.pone.0113728 (PMC4239102; doi:10.1371/journal.pone.0113728)
Supplement: Table S1 — Active substances and their doses used for exposures through thoracic topical exposure. ng a.s./bee: nanogram of active substance per bee. Nr: Number of replicates per dose. ABA = Abamectin, ACE = Acetamiprid, CHL = Chlorpyrifos-ethyl, CLO = Clothianidin, CYF = Cyfluthrin, CYP = Cypermethrin, DEL = Deltamethrin, DIM = Dimethoate, ESF = Esfenvalerate, IMI = Imidacloprid, LAM = Lambda-cyhalothin, PRO = Prochoraz, TAU = Tau-fluvalinate, THI = Thiacloprid, TMX = Thiamethoxam. (DOCX) [file pone.0113728.s016.docx]

**Table S1. Active substances and their doses used for exposures through thoracic topical exposure**

| **Group** | **N_r_** | **Doses (ng a.s./bee)** | | | | | | | | | | | | | | |
| --- | --- | --- | --- | --- | --- | --- | --- | --- | --- | --- | --- | --- | --- | --- | --- | --- |
|  |  | **ABA** | **ACE** | **CHL** | **CLO** | **CYF** | **CYP** | **DEL** | **DIM** | **ESF** | **IMI** | **LAM** | **PRO** | **TAU** | **THI** | **TMX** |
| Control | 11 | 0 | 0 | 0 | 0 | 0 | 0 | 0 | 0 | 0 | 0 | 0 | 0 | 0 | 0 | 0 |
| 1 | 15 | 0.5 | 500 | 20 | 1 | 1 | 15 | 20 | 50 | 5 | 5 | 5 | 1 000 | 500 | 1 000 | 5 |
| 2 | 15 | 1 | 1 000 | 30 | 5 | 4 | 30 | 30 | 75 | 25 | 10 | 10 | 3 000 | 1 000 | 5 000 | 10 |
| 3 | 15 | 1.5 | 2 000 | 40 | 10 | 8 | 50 | 60 | 100 | 50 | 25 | 20 | 5 000 | 1 500 | 10 000 | 25 |
| 4 | 9 | 2 | 3 000 | 50 | 20 | 12 | 75 | 90 | 125 | 75 | 50 | 40 | 7 000 | 2 000 | 20 000 | 40 |
| 5 | 9 | 2.5 | 4 000 | 60 | 30 | 16 | 100 | 120 | 150 | 100 | 75 | 60 | 8 000 | 2 500 | 30 000 | 50 |
| 6 | 9 | 3 | 5 000 | 70 | 35 | 20 | 120 | 150 | 175 | 150 | 100 | 75 | 9 000 | 3 000 | 40 000 | 60 |
| 7 | 6 | 3.5 | 7 500 | 80 | 40 | 40 | 160 | 180 | 200 | 200 | 200 | 100 | 10 000 | 3 500 | 50 000 | 80 |
| 8 | 6 | 4 | 10 000 | 90 | 60 | 60 | 200 | 210 | 225 | 250 | 400 | 150 | 20 000 | 4 000 | 60 000 | 100 |
| 9 | 6 | 5 | 12 500 | 100 | 75 | 80 | 250 | 250 | 250 | 300 | 600 | 200 | 25 000 | 6 000 | 80 000 | 200 |
| 10 | 6 | 6 | 15 000 | 150 | 100 | 100 | 300 | 300 | 300 | 400 | 800 | 300 | 30 000 | 12 000 | 100 000 | 250 |

ng a.s./bee: nanogram of active substance per bee.

Nr: Number of replicates per dose.

ABA=Abamectin, ACE=Acetamiprid, CHL=Chlorpyrifos-ethyl, CLO=Clothianidin, CYF=Cyfluthrin, CYP=Cypermethrin, DEL=Deltamethrin, DIM=Dimethoate, ESF=Esfenvalerate, IMI=Imidacloprid, LAM=Lambda-cyhalothin, PRO=Prochoraz, TAU=Tau-fluvalinate, THI=Thiacloprid, TMX=Thiamethoxam.
