# Supplementary material for: A Pragmatic Approach to Assess the Exposure of the Honey Bee (Apis mellifera) When Subjected to Pesticide Spray
Source: PLoS One. 2014 Nov 20;9(11):e113728. doi: 10.1371/journal.pone.0113728 (PMC4239102; doi:10.1371/journal.pone.0113728)
Supplement: Table S2 — Classification of the 20 active substances by their revisited HQs for different values of exposure surface areas. An active substance was classified with a HQ≥1 if at least the revisited HQ of one of the two scenarios (Table 5) were equal to or higher than this value. Lower C.I.: Lower limit of the Confidence Interval at 95%. Upper C.I.: Upper limit of the Confidence Interval at 95%. (DOCX) [file pone.0113728.s017.docx]

**Table S2.** **Classification of the 20 active substances by their revisited HQs for different values of exposure surface areas**

|  | **Number of active substances** **classified by categories** | | |
| --- | --- | --- | --- |
|  | **Lower C.I. (0.89 cm²)** | **Mean (1.05 cm²)** | **Upper C.I. (1.21 cm²)** |
| HQ ≥ 1 | 12 | 12 | 12 |
| HQ < 1 | 8 | 8 | 8 |

An active substance was classified with a HQ ≥ 1 if at least the revisited HQ of one of the two scenarios (Table 5) were equal to or higher than this value.

Lower C.I.: Lower limit of the Confidence Interval at 95%.

Upper C.I.: Upper limit of the Confidence Interval at 95%.
